# Automated Coregistered Segmentation for Volumetric Analysis of Multiparametric Renal MRI

**DOI:** 10.1002/mrm.70288

**Published:** 2026-02-04

**Authors:** Aya Ghoul, Cecilia Liang, Isabelle Loster, Lavanya Umapathy, Bernd Kühn, Petros Martirosian, Ferdinand Seith, Sergios Gatidis, Thomas Küstner

**Affiliations:** ^1^ Medical Image and Data Analysis (MIDAS.Lab), Department of Interventional and Diagnostic Radiology University Hospital of Tuebingen Tuebingen Germany; ^2^ Department of Interventional and Diagnostic Radiology University Hospital of Tuebingen Tuebingen Germany; ^3^ Center for Advanced Imaging Innovation and Research (CAI2R), Department of Radiology New York University Grossman School of Medicine New York New York USA; ^4^ Siemens Healthineers AG Erlangen Germany; ^5^ Department of Radiology Stanford University Stanford California USA

**Keywords:** contrastive learning, deep learning, image registration, multiparametric renal MRI, segmentation

## Abstract

**Purpose:**

This study aims to develop and evaluate a fully automated deep learning‐driven postprocessing pipeline for multiparametric renal MRI, enabling accurate kidney alignment, segmentation, and quantitative feature extraction within a single efficient workflow.

**Methods:**

Our method has three main stages. First, a segmentation network delineates renal structures in high‐contrast images. Next, a deep learning‐based pairwise image registration algorithm maps the multiparametric image series to a common target and transfers the predicted annotations between the multiparametric images. Finally, clinically relevant quantitative parameters are extracted through region‐specific assessment of renal structure and function based on the aligned and segmented multiparametric data. We used five‐fold cross‐validation to compare the segmentation outcomes and extracted features with manual analyses in 24 patients with prostate cancer or neuroendocrine tumors and 10 healthy subjects, each undergoing repeated scans.

**Results:**

Our automated pipeline achieved high agreement with expert kidney segmentation while delivering significant alignment improvements through registration. Volumetric analysis showed a strong correlation (*r* > 0.9) with manual results, and feature extraction demonstrated high intraclass correlation coefficients with minimal bias. The complete processing pipeline, encompassing coregistration, segmentation, and feature extraction, required approximately 15 s per scan from raw input to final quantitative output.

**Conclusion:**

The study establishes a reliable automated pipeline for renal multiparametric MRI postprocessing. The achieved accuracy and efficiency can support improved diagnosis and treatment planning for patients with kidney disease.

## 1 Introduction

Functional magnetic resonance imaging (fMRI) provides non‐invasive structural and functional markers that facilitate the prediction and longitudinal monitoring of renal dysfunction in a single scan session [1, 2, 3]. Multi‐parametric renal fMRI protocols provide a comprehensive evaluation of key physiological processes, including renal diffusion [4, 5], hemodynamics [6, 7], oxygenation [8, 9], and microstructure organization and function [10, 11]. Robust analysis workflows are required to ensure reproducible quantitative assessments and maximize the information derived from the listed protocols. Accurate segmentation of renal tissue constitutes a fundamental step in this process. However, manual delineation is not only time‐consuming and subject‐dependent, but also hindered by the inherent anatomical variability, physiological motion, and patient positioning [12, 13, 14]. The segmentation task is even more demanding in low‐contrast imaging modalities, such as diffusion‐weighted imaging, where distinguishing the renal cortex from the medulla becomes challenging.

Automated methods have been explored to address the limitations of manual segmentation. Early attempts [15, 16, 17], relying on techniques like clustering and contour detection, were often limited by overlapping gray‐level intensities. More recently, deep learning models [18, 19, 20, 21] have emerged as a promising alternative, achieving state‐of‐the‐art performance without manual intervention or handcrafted features. Self‐supervised learning [22, 23], particularly contrastive learning, has provided a robust network initialization and improved feature representation to enhance segmentation accuracy [24, 25, 26].

In addition to image segmentation, comprehensive analysis in longitudinal and multi‐sequence imaging studies necessitates establishing the pixel‐to‐pixel correspondence between the multi‐parametric images [27]. This enables tracking morphological changes [28], facilitates parameter mapping [29], and provides a solid basis for evaluating treatment response [30]. Although rigid transformations [31, 32] are computationally efficient, they often fail to capture the complex deformations that kidneys undergo, such as shearing and size variations. Deformable image registration methods [33, 34, 35, 36, 37] offer a more accurate non‐rigid motion representation. To address the challenge of varying image contrast, groupwise registration to a common mean space has been adopted [38]. Further improvements have been achieved by incorporating contrast‐independent features into similarity metrics [39, 40], and low‐rank minimization techniques [41]. Yet, conventional approaches remain computationally intensive and sensitive to initialization, limiting their practical application. Recent advancements in deep learning have introduced an alternative solution with improved efficiency [41, 42, 43, 44, 45], which is relevant for clinical practice and research.

Accurate segmentation is essential for delineating anatomical structures, while robust registration ensures spatial alignment across different parametric images and time points. Automating these processes can mitigate inter‐observer variability, increase data processing throughput, and facilitate large‐scale cohort studies. Nevertheless, a significant translational gap persists between developing automated analysis methods and their integration into clinical routine. To this end, we introduce a fully automated deep learning‐based pipeline that handles multi‐parametric data efficiently to generate clinically reliable measurements. The proposed method is structured in three stages: First, a segmentation network labels kidney structures in high‐tissue‐contrast images, such as Dixon images. We apply contrastive learning [24] to mitigate the limited available annotations and establish a strong initial prior, which is then fine‐tuned for the downstream task of image segmentation. Second, a pairwise image registration maps the multi‐parametric images to a unified spatial space. Finally, quantitative parameters are extracted through comprehensive feature analysis of the obtained co‐registered and segmented multi‐parametric data.

Our key contributions are: (1) We developed a deep learning‐based post‐processing workflow that can provide automated standardized processing for renal multi‐parametric MRI within 15 s. The performance of the pipeline was evaluated through five‐fold cross‐validation on a cohort of 24 patients with prostate cancer or neuroendocrine tumors, and 10 healthy subjects across repeated scans, (2) We demonstrated that image registration enables the propagation of segmentation masks from high‐contrast to low‐contrast images, resulting in reliable quantitative measurements comparable to those of experienced radiologists, and (3) We benchmarked our method against manual analysis in image segmentation, volumetric analyses, and feature extraction.

## 2 Methods

### 2.1 Data Acquisition

Scanning was performed on a 3T MRI scanner (MAGNETOM Prisma^fit^, Siemens Healthcare AG, Erlangen, Germany) without contrast media, using an 18‐channel matrix‐array coil in combination with a 12‐channel spine‐array coil. 24 patients with prostate cancer or neuroendocrine tumors (25% female, 71 ± 9 years) and 10 healthy subjects (50% female, 31 ± 4 years) were recruited. A total of 76 multi‐parametric scans were acquired from 34 participants as part of a longitudinal study. Written informed consent was obtained from all subjects for the examination and the publication of the results.

The multi‐parametric renal MRI scan protocol [46] consisted of six contrasts: (1) $T_{1}$ mapping applying a variable flip angle (VFA) method using a volumetric interpolated breath‐hold examination (VIBE) sequence, (2) $T_{2}$ mapping with a $T_{2}$ prep turbo fast low angle shot (TFL), (3) water‐fat MR images obtained using a two‐point Dixon‐Method with a VIBE sequence [47], (4) renal blood flow (RBF) mapping using a research pseudo‐continuous arterial spin labeling (PCASL) sequence [48], (5) $T_{2}^{*}$ based on blood oxygen level dependent (BOLD) imaging with a multi‐echo spoiled gradient echo (mGRE) sequence, and (6) apparent Diffusion Coefficient (ADC) mapping using intravoxel incoherent motion diffusion‐weighted imaging prototype sequence (DWI‐IVIM) with a reduced field‐of‐view. Variations in resolution and matrix size across contrasts are summarized in Table 1, along with further protocol details.

**TABLE 1:** Acquisition parameters of the renal multiparametric MR protocol.

	Dixon	$T_{1}$	$T_{2}$	BOLD/$T_{2}^{*}$	RBF	ADC
Sequence	VIBE	VFA	$T_{2}$ prep TFL	mGRE	PCASL	DWI‐IVIM
TR (ms)	3.95	3.5	5000	133	6000–7400	1500
TE (ms)	1.23/2.46	1.24	2.59	2.46–46.76	27.46	55.0
Flip angle (°)	9	2.10	8	40	90 180	90 180
FOV (mm^3^)	380 × 380 × 96	380 × 380 × 96	380 × 380 × 8	380 × 380 × 54	380 × 380 × 144	380 × 380 × 72
Matrix size	256 × 205 × 32	256 × 205 × 32	256 × 205 × 1	256 × 205 × 12	96 × 48 × 24	192 × 96 × 24
Resolution (mm^3^)	1.5 × 1.5 × 3	1.5 × 1.5 × 3	1.5 × 1.5 × 8	4 × 4 × 6	1.5 × 1.5 × 3	1.5 × 1.5 × 3
# Subjects scans	76	68	76	76	76	76
# Patient scans	58	52	58	58	56	58

The left and right renal cortex and total kidney volume, excluding the renal pelvis, were manually segmented from surrounding tissues across all available image slices by a radiologist with six years of experience in fMRI analysis. The medullary components were obtained by subtracting the segmentation masks of the cortex from the total kidney segmentation. Manual segmentation was performed using the NORA Medical Imaging Platform [49] and required, on average, 2 h per scan. For evaluation of inter‐reader agreement, the $T_{1}$ images were additionally segmented by two experienced radiologists.

### 2.2 Postprocessing Pipeline for Multiparametric Renal fMRI

**FIGURE 1:**
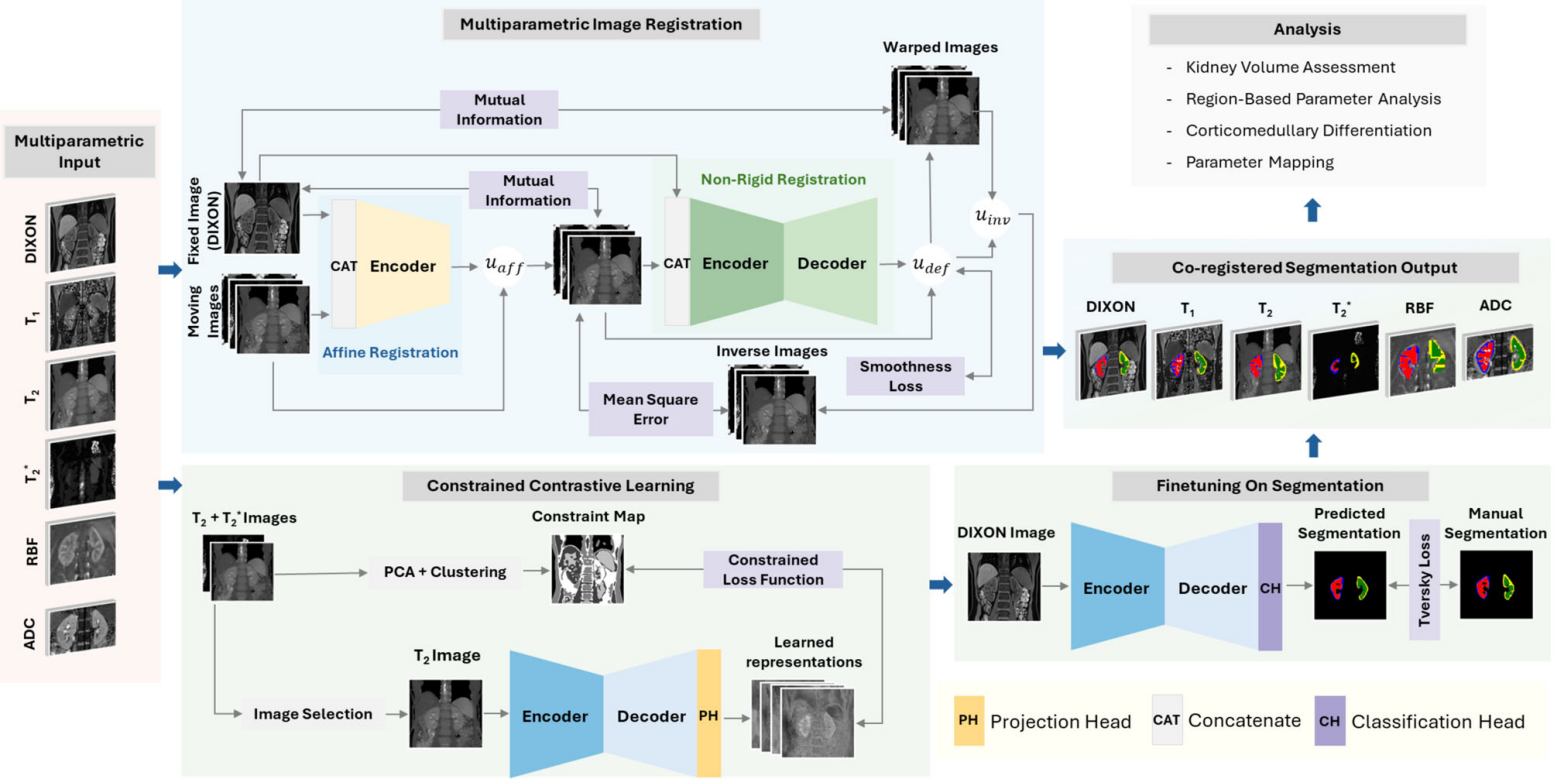
The proposed post‐processing pipeline for multi‐parametric renal MRI. Contrastive learning [24] enables the segmentation model to learn discriminative local representations to be fine‐tuned later to generate labels for high‐contrast images. These labels are then propagated to low‐contrast images using motion estimation from the image registration network. The resulting co‐registered and segmented dataset enables quantitative feature extraction, including kidney volume estimation and region‐specific parameter analysis.

We propose a post‐processing workflow for renal fMRI (Figure 1), designed for efficiency and robustness despite limited labeled data. We use two separate deep learning models: (1) a segmentation network initialized with contrastive learning to provide a strong starting point for training on scarce annotations, and (2) a pairwise image registration network. Initially, high‐contrast images (Dixon, $T_{1}$, $T_{2}$, $T_{2}^{*}$, ADC) are segmented using the segmentation network. Multi‐parametric scans are also aligned to the Dixon images, which serve as a common reference space. This enables the propagation of Dixon‐based annotations to other contrasts, improving segmentation accuracy for challenging contrasts such as RBF. The resulting aligned and segmented images are then used for downstream analyses.

#### 2.2.1 The U‐Net Backbone

The segmentation and registration models use a 2D U‐Net backbone with an encoding depth of 5, corresponding to 5 resolution levels and 4 downsampling steps [50]. A 2D design is adopted due to anisotropic resolution and the limited through‐plane coverage of our database. The first encoding level has 3 × 3 convolutional layers, starting with 16 kernels and doubling after each 2 × 2 max‐pooling downsampling, culminating in 256 kernels at the bottleneck. The decoder mirrored the kernel count of the encoder, employing 2×2 nearest‐neighbor upsampling. Leaky rectified linear unit (ReLU) activation with a slope of 0.01 and batch normalization were applied after each convolutional operation. Residual connections are incorporated between all encoder and decoder blocks of the same spatial resolution.

#### 2.2.2 Image Segmentation

##### 2.2.2.1 Constrained Contrastive Learning

The contrast in MR images is intrinsically linked to underlying tissue properties (e.g., $T_{1}$/$T_{2}$ relaxation times) and image acquisition parameters. We selected $T_{2}$ and $T_{2}^{*}$ images to learn discriminative local representations due to their sensitivity to intrinsic tissue characteristics, such as water content, perfusion, and oxygenation, which provide strong cues for distinguishing renal cortex and medulla and pathological areas [24]. These contrasts produce feature embeddings that are well‐separated in the learned representation space, resulting in clear cluster formation in Uniform Manifold Approximation and Projection (UMAP) maps (Figure S1). Although other contrasts were excluded during pre‐training, the learned embeddings generalize effectively, providing meaningful guidance for segmentation across these images as well.

We use a constrained contrastive learning (CCL) strategy to encourage local feature consistency and enhance separability of renal sub‐regions. $T_{2}$ and $T_{2}^{*}$ images are processed in two parts. First, pixel‐wise principal component analysis (PCA) is applied to reduce redundancy, retaining P=20 principal components. These components are then clustered by k‐means (*K* = 4) to generate a constraint map, which encodes pixel‐level similarity information and enforces that voxels with comparable intensity and texture are embedded closely in feature space (Figure S2). Second, a non‐linear projection head (PH) is appended to the encoder/decoder U‐Net network to embed the features into a representation space of N=64 channels during pre‐training, capturing the underlying image structure and similarity relationships encoded by the constraint map.

The constrained loss [24] operates on a patch‐level between the constraint map and the learned representations. We randomly sample 100 patches of size *p* = 4 × 4. For each patch, a label is assigned based on the dominant cluster within the corresponding patch in the constraint map. The loss utilizes an L2‐normalized cosine similarity with a temperature τ of 0.1 to minimize the feature distance between patches sharing the same dominant cluster label. This encourages the model to generate similar representations for patches from similar anatomical regions that share underlying $T_{2}$ and $T_{2}^{*}$ properties. As a result, the learned patch embeddings create a representational space that embodies patch‐level similarities derived from the constraint map.

To support our design choices, we provide UMAP visualizations of the normalized learned feature space (Figure 3). These embeddings confirm that the selected hyperparameters yield well‐separated clusters across different embedding dimensions, patch sizes, and temperatures, supporting the effectiveness of our CCL configuration. We optimized the contrastive loss over 200 epochs with an initial learning rate of 0.001 using an Adam optimizer and a batch size of 8.

##### 2.2.2.2 Fine‐tuning

Following contrastive pre‐training, the U‐Net network is fine‐tuned for segmentation on all multi‐parametric images. We replace the non‐linear projection head with a softmax function to generate posterior probabilities for each class. We train two segmentation networks: one for 3‐class segmentation (left/right kidney and background) and another for 5‐class segmentation (left/right medulla, cortex, and background).

Tversky loss was employed to train the two models operating on full‐sized images, with the α and β penalties controlling false positives and false negatives set to 0.3 and 0.7, respectively [51]. We included label weighting to mitigate class imbalance due to the background. A weight $w_{c}$ was assigned to each class c, defined as inversely proportional to its frequency in the label [52].

During fine‐tuning, data augmentation was performed on the fly to learn transformation invariant features, including rotation, cropping, scaling, elastic transformations, and additive brightness and contrast. We used an Adam optimizer with an initial learning rate of 0.01, a polynomial learning rate decay schedule [53], and a Nesterov momentum of 0.99. Training proceeded for 500 epochs, with each epoch consisting of 250 iterations. The Dice score served as the validation metric.

#### 2.2.3 Multiparametric Image Registration

Multi‐parametric images were mapped to a common target space via a pairwise registration network. Dixon images were used as the reference due to their high tissue contrast and well‐defined anatomical landmarks. The registration network uses a two‐stage approach: an initial affine transformation for coarse alignment, followed by a deformable network for fine‐grained adjustments.

##### 2.2.3.1 Affine Image Registration

The affine network captures the global geometric transformation using the encoder part of the U‐Net architecture as a feature extractor. These features are passed through a 1 × 1 convolution, 2 × 2 max pooling, and flattened before being fed into two fully connected layers of 16 and 6 units sequentially. The output represents the parameters of the affine transformation matrix, which is converted into a dense displacement field. The input includes the Dixon image $I_{\mathrm{fix}}$ and a sequence of N=5 multi‐parametric moving images $I_{N}$ consisting of $T_{1}$, $T_{2}$, $T_{2}^{*}$, RBF, and ADC images. The network learns N motion estimates ($u_{\mathrm{aff}}$) that map the moving images to the Dixon image.

We used the normalized mutual information ${ℒ}_{\mathrm{NMI}}$ to measure the similarity between the fixed images $I_{\mathrm{fix}}$ and the warped images $u_{\mathrm{aff}}\circ I_{N}$ despite contrast variations [54]:

(1) $${ℒ}_{\mathrm{NMI}}=-\frac{1}{N}\sum_{i=1}^{N}\frac{2\mathrm{MI}\left(I_{\mathrm{fix}},u_{\mathrm{aff},i}\circ I_{i}\right)}{H\left(I_{\mathrm{fix}}\right)+H\left(u_{\mathrm{aff},i}\circ I_{i}\right)}$$

where MI and H denote the mutual information and the entropy, respectively. Moreover, we used the Dice score ${ℒ}_{\mathrm{DSC}}$ as an additional guiding loss to quantify the volume overlap for the left and right kidneys. The total training loss for the affine network is:

(2) $${ℒ}_{\text{affine}}={ℒ}_{\mathrm{NMI}}+{ℒ}_{\mathrm{DSC}}$$

##### 2.2.3.2 Non‐rigid Image Registration

The non‐rigid registration network consists of the previously described U‐Net backbone followed by two convolutional layers, with 16 and 2 filters, respectively, separated by a ReLU activation function at the output. These additional layers reduce the channel dimension to two, corresponding to the spatial coordinates of the deformation field. We incorporate the pre‐trained, frozen affine transformation network into the non‐rigid registration process. The affine‐transformed multi‐parametric images $u_{\mathrm{aff}}\circ I_{N}$, along with the fixed images $I_{\mathrm{fix}}$, are stacked and serve as the input to the non‐rigid registration network. This initial affine transformation minimizes discrepancies and provides a preliminary, rough alignment with the fixed image. The output encompasses N deformable motion estimates $u_{\mathrm{def}}$ that map the affine‐corrected moving images ($u_{\mathrm{aff},i}\circ I_{i}$) to the fixed image ($I_{\mathrm{fix}}$). The inverse transformation $u_{\mathrm{inv}}$ that aligns the fixed image ($I_{\mathrm{fix}}$) with the affine‐corrected moving images ($u_{\mathrm{aff},i}\circ I_{i}$) is also determined using a fixed‐point method [55] to enforce bi‐directional consistency [56].

We used the normalized mutual information ${ℒ}_{\mathrm{NMI}}$ as a similarity metric. To enforce realistic estimates, we introduced regularization losses: the Dice loss ${ℒ}_{\mathrm{DSC}}$ to promote accurate overlap, and a smoothness loss ${ℒ}_{\text{smoothness}}$ to penalize abrupt local deformation changes. ${ℒ}_{\text{smoothness}}$ is defined as:

(3) $${ℒ}_{\text{smoothness}}=\frac{1}{N}\sum_{i=1}^{N}∥\nabla u_{{\mathrm{def}}_{i}}{∥}_{1}$$

where ∇ denotes the spatial gradient operator. Furthermore, we incorporated a bi‐directional warping consistency loss ${ℒ}_{\text{consistency}}$. This loss calculates the mean squared error (MSE) between the parametric images $I_{i}$ and their transformations by both forward and backward warping $I_{\mathrm{bi}-\text{warp},i}=u_{\mathrm{inv}}\circ u_{\mathrm{def}}\circ I_{i}$, using the backward $u_{\mathrm{inv}}$ and forward non‐rigid deformation $u_{\mathrm{def}}$:

(4) $${ℒ}_{\text{consistency}}=\frac{1}{N}\sum_{i=1}^{N}∥I_{i}-I_{\mathrm{bi}-\text{warp},i}{∥}_{2}$$

${ℒ}_{\text{consistency}}$ promotes smooth and physically plausible deformations by discouraging irregular transformations. The total training loss is given as:

(5) $${ℒ}_{\text{total}}={ℒ}_{\mathrm{NMI}}+\lambda_{1}{ℒ}_{\mathrm{DSC}}+\lambda_{2}{ℒ}_{\text{smoothness}}+\lambda_{3}{ℒ}_{\text{consistency}}$$

where $\lambda_{1}=1$, $\lambda_{2}=0.1$, and $\lambda_{3}=100$ are the weights assigned to the losses, optimized by performing a parameter search to maximize the Dice score on the validation dataset. The training of both networks was performed with an Adam optimizer with an initial learning rate of 0.001 and a polynomial learning rate decay schedule [53]. Training proceeded for 250 epochs, with each epoch consisting of 1000 iterations, for a batch size of 8.

#### 2.2.4 Feature Extraction

Our segmentation network generates masks of the kidney parenchyma for all contrasts. In high‐contrast images (Dixon, $T_{1}$, $T_{2}$, $T_{2}^{*}$), cortex and medulla are also segmented. The Dixon‐derived segmentation is propagated to lower‐contrast perfusion and diffusion‐weighted images, which are inherently difficult to segment directly. The resulting co‐aligned and segmented multi‐parametric dataset enables the extraction of quantitative imaging biomarkers for renal function characterization. Total kidney, cortex, and medulla volumes are calculated from coronal slices of the Dixon images. Region‐specific mean signal intensities and physiological parameters are extracted by applying binary masks of the kidney parenchyma, cortex, and medulla separately to each parametric map.

### 2.3 Training, Validation, and Testing

All data were resampled to 1.5×1.5×3 mm^3^ using third‐order spline interpolation for images and nearest‐neighbor for masks. Coronal slices, which served as the input to the network, were zero‐padded to 256×256 to standardize dimensions. Intensities were clipped to the [0.05,99.5] percentile and z‐score normalized per subject and contrast. During testing, predicted masks were cropped to the original matrix size and resampled to native resolution with nearest‐neighbor interpolation.

Training and inference were conducted on a single Nvidia V100 GPU. Training durations were as follows: 48 h for pre‐training, 24 h for each segmentation network, 24 h for affine registration, and 36 h for deformable registration. The processing pipeline, which includes co‐registration, segmentation, and feature extraction, was implemented to run efficiently on GPU and CPU environments. Parallelization on multiple CPU cores (6 cores of an AMD EPYC 7502 CPU) was enabled to support CPU‐only execution.

We performed five‐fold cross‐validation, splitting 32 subjects into separate training (80%, 19 patients and 7 healthy volunteers) and test (20%, 4 patients and 2 healthy volunteers) subsets. In each fold, one of the five subsets served as the test set, while the other four subsets were used for training. The remaining subjects (one patient and one healthy volunteer) were designated exclusively for validation and hyperparameter tuning. Due to repeated scan sessions, the effective multiparametric sample size per subject ranged from 2 to 4. Parametric images with incomplete measurements or insufficient quality were excluded. The number of available scans for each contrast is detailed in Table 1.

### 2.4 Experiments

#### 2.4.1 Segmentation Evaluation

We evaluated the accuracy of the segmentation by comparing the predicted and manual segmentation masks of each contrast using the Dice similarity coefficient (DSC) and the Hausdorff distance (HDD). HDD, measured in millimeters, quantifies the maximum distance between the boundaries of automated and manual segmentation, with 0 mm indicating perfect overlap. Both metrics were calculated for the left and right kidney parenchyma, cortex, and medulla. An ablation study was conducted to investigate the effect of contrastive learning pre‐training. The effect of scarce labeled data was investigated by fine‐tuning a pre‐trained model on subsets of 6, 12, and 26 subjects (the full training set). Segmentation performance was then compared with a randomly initialized model using a fixed test fold of 6 subjects. Inter‐reader agreement for $T_{1}$ images was also assessed between three experienced radiologists using DSC and HDD scores.

#### 2.4.2 Registration Evaluation

To assess the registration performance, we evaluated image similarity and landmarks overlap at three stages: before registration, after affine registration, and after affine and non‐rigid registration. We reported the normalized mutual information $D_{\mathrm{NMI}}$ and the PCA‐based deformation index ($D_{\mathrm{PCA}}$) [38]. $D_{\mathrm{PCA}}$ represents the ratio of the top‐K eigenvalues to the total sum of eigenvalues in the correlation matrix. A lower $D_{\mathrm{PCA}}$ value indicates improved registration performance. The segmentation masks of the individual contrasts were aligned by the registration network. For each contrast, we quantified the Dice similarity coefficient (${\mathrm{DSC}}_{\text{warp}}$) between the warped masks and the ground truth manual mask.

The percentage of non‐positive Jacobian determinant values was reported to evaluate the smoothness and anatomical plausibility of the motion estimates. Negative values indicate folding or unrealistic deformations, and values approaching zero suggest near‐singular transformations. A 0% non‐positive Jacobian value is preferred to ensure diffeomorphism. Furthermore, we conducted a qualitative assessment by visualizing quiver plots and warped segmentation masks overlaid on the moving images.

We conducted a comparison against two alternative registration architectures, to validate the suitability of a 2D strategy for anisotropic renal MRI: (1) a 2.5D slice‐stacking U‐Net incorporating five adjacent slices (current slice ±2), and (2) a full 3D U‐Net trained on entire volumes. All models were trained using identical data partitions, intensity normalization, augmentation strategies, and loss functions to ensure methodological parity. For 2.5D, adjacent slices were stacked along the channel dimension, but only the central slice was used for loss computation, allowing direct comparison to the 2D output.

#### 2.4.3 Baseline Comparisons

For a comprehensive comparison, we benchmarked our method against established baselines in segmentation and registration. Our segmentation method was compared with the widely used nnU‐Net [19], following its self‐configuring protocol. 2D and 3D full‐resolution architectures were trained with five‐fold cross‐validation. For the 3D configuration, input patches of size 32×256×224 voxels were sampled from the original median image shape of 32×254×254. The 2D configuration used 256×256 coronal slices. Both architectures employed U‐Net variants with instance normalization and LeakyReLU activations. The models were trained for 1000 epochs using a stochastic gradient descent optimizer with momentum (μ=0.99), combined Dice‐Cross‐Entropy loss, and standard data augmentation. Final segmentation masks were generated by ensembling predictions from both architectures across all cross‐validation folds.

For registration baseline comparison, we compared the proposed registration strategy against three conventional image registration methods: SyN [35], LDDMM [36], and Elastix [37]. Hyperparameters were tuned via random search to maximize the kidney segmentation Dice score on the validation set. For SyN registration, the Mattes Mutual Information metric [57] with Gaussian smoothing was optimized for 250 iterations at each of the five resolution levels. LDDMM used normalized Mutual Information [54] with motion regularization and a kernel size of 3. The matching and regularization terms were weighted 4 and 10, respectively, over 500 iterations. A four‐level multi‐resolution scheme was adopted in Elastix with filter sizes of 32, 16, 8, and 4. The Parzen Window Mutual Information metric [57] was optimized over 4096 randomly sampled spatial coordinates for 1000 iterations per level.

#### 2.4.4 Feature Extraction Agreement

We investigated the agreement between the predicted and manual total kidney, medulla, and cortex volumes using regression plots and modified Bland–Altman plots. For different contrast maps, we separately compared the extracted feature mean values from manual and predicted masked maps using our framework for the kidney, cortex, and medulla. Additionally, the mean RBF in the kidneys obtained with our proposed 2D registration method was compared against 2.5D and 3D registration baselines. Agreement between manual and predicted features was quantified using bias and intra‐class correlation coefficient (ICC) with 95% confidence intervals. We reported the mean and standard deviation (SD) of all evaluated metrics, averaged across subjects. Significance was established at a threshold of p<0.05, adjusted for multiple comparisons.

## 3 Results

### 3.1 Automated Segmentation Performance

**FIGURE 2:**
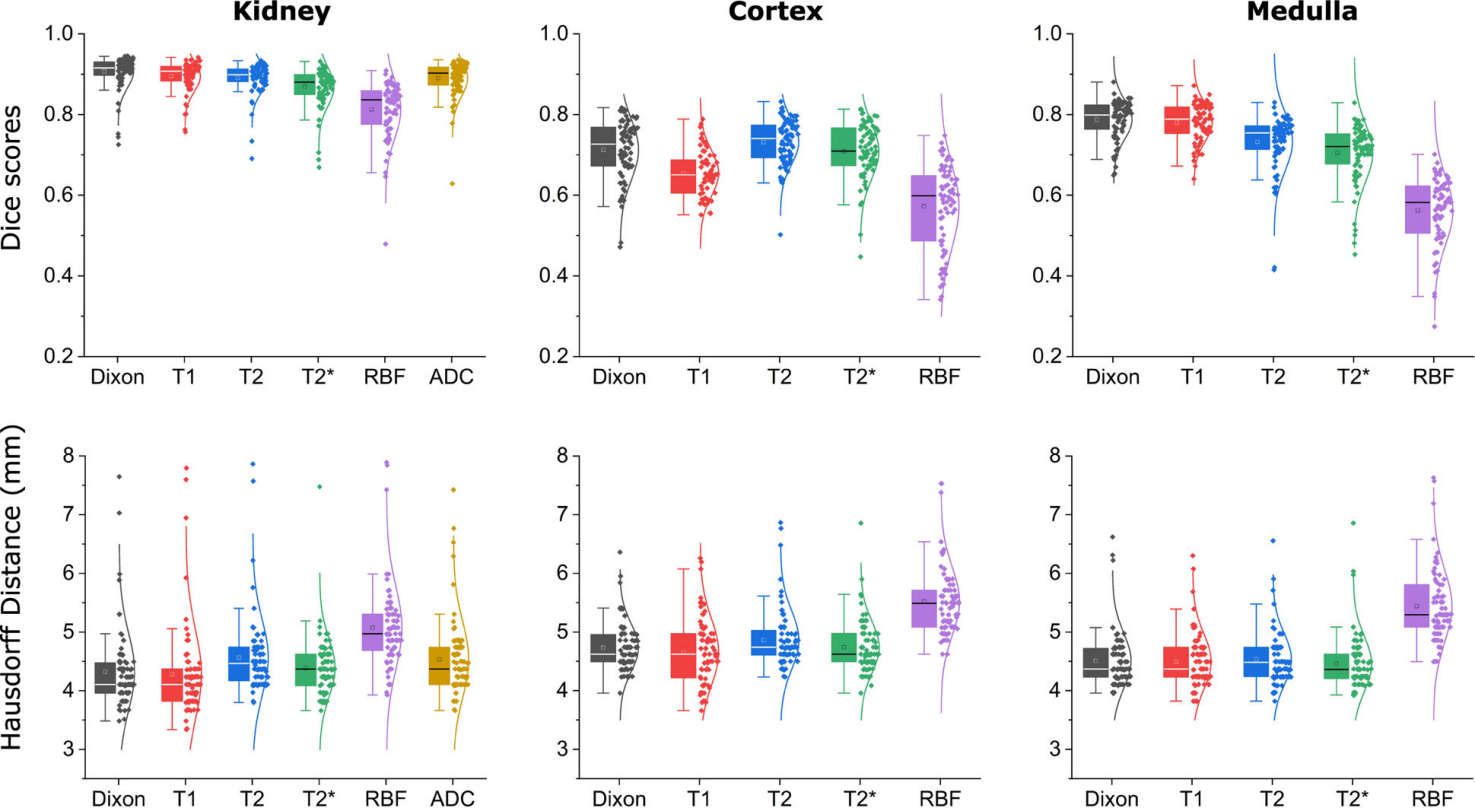
Comparison of Dice scores quantifying the overlap between segmentation masks generated by our model and expert manual segmentations. Metrics are presented for Dixon imaging, $T_{1}$‐mapping, $T_{2}$‐mapping, $T_{2}^{*}$ imaging, RBF perfusion, and diffusion‐weighted imaging (ADC), using five‐fold cross‐validation across the entire cohort. Each dot corresponds to a single scan volume. Results are reported for the kidney parenchyma, the cortex, and the medulla. The analysis demonstrates that high‐contrast images, including Dixon, $T_{1}$, $T_{2}$, and $T_{2}^{*}$ images, produce superior segmentation results compared to RBF.

The quantitative five‐fold cross‐validation outcomes for automated segmentation are shown in Figure 2 for various parametric images, including Dixon, $T_{1}$ mapping, $T_{2}$ mapping, $T_{2}^{*}$, RBF, and ADC. The DSC and HDD are presented for three anatomical regions: the kidney parenchyma, cortex, and medulla. ADC results for the cortex and medulla are omitted due to insufficient contrast for manual segmentation.

The cross‐validation results indicated high segmentation accuracy for whole kidney volumes across the imaging sequences, with DSC values consistently above 0.8. Dixon achieved the highest DSC (0.91±0.04), followed by $T_{1}$ mapping (0.9±0.01), $T_{2}$ mapping (0.89±0.04), ADC (0.89±0.04), and $T_{2}^{*}$ (0.87±0.05), all demonstrating high agreement with manual segmentation. RBF yielded the lowest DSC (0.81±0.07) and the highest variability. HDD measurements further support these trends, with the lowest values observed for $T_{1}$ mapping (4.14±0.39) and Dixon (4.32±0.67). $T_{2}$ mapping (4.57±0.65), $T_{2}^{*}$ (4.39±0.5), and ADC (4.53±0.65) showed slightly higher HDD values, suggesting minor segmentation deviations. RBF had the highest HDD (5.07±0.71) and greatest variability, consistent with the DSC outcomes.

Segmenting the cortex and medulla proved more challenging than whole kidney segmentation, as evidenced by lower DSC values and higher HDD scores. Despite extensive consensus, inter‐reader agreement in manual $T_{1}$ segmentation was also higher for total kidney volumes (${\mathrm{DSC}}_{\text{kidney}}=0.74-0.86$, ${\mathrm{HDD}}_{\text{kidney}}=4.42-4.48$) than cortical (${\mathrm{DSC}}_{\text{cortex}}=0.61-0.76$, ${\mathrm{HDD}}_{\text{cortex}}=4.68-5.28$) and medullary (${\mathrm{DSC}}_{\text{medulla}}=0.56-0.77$, ${\mathrm{HDD}}_{\text{medulla}}=4.71-5.56$) delineation, underscoring the inherent difficulty of cortex and medulla annotation. Among all imaging sequences, Dixon provided the best segmentation performance (${\mathrm{DSC}}_{\text{cortex}}:0.72\pm 0.08$ and ${\mathrm{DSC}}_{\text{medulla}}:0.77\pm 0.08$, ${\mathrm{HDD}}_{\text{cortex}}:4.73\pm 0.41$ and ${\mathrm{HDD}}_{\text{medulla}}:4.51\pm 0.47$). $T_{1}$, $T_{2}$ and $T_{2}^{*}$ sequences yielded DSC scores exceeding 0.7. In contrast, RBF resulted in the lowest segmentation accuracy (${\mathrm{DSC}}_{\text{cortex}}:0.57\pm 0.11$ and ${\mathrm{DSC}}_{\text{medulla}}:0.56\pm 0.08$, ${\mathrm{HDD}}_{\text{cortex}}:5.52\pm 0.58$ and ${\mathrm{HDD}}_{\text{medulla}}:5.44\pm 0.62$). Cortex and medulla segmentation exhibited more frequent and severe outliers than the whole kidney, particularly in RBF images, with Dice scores below 0.5 and Hausdorff distances exceeding 6 mm.

**FIGURE 3:**
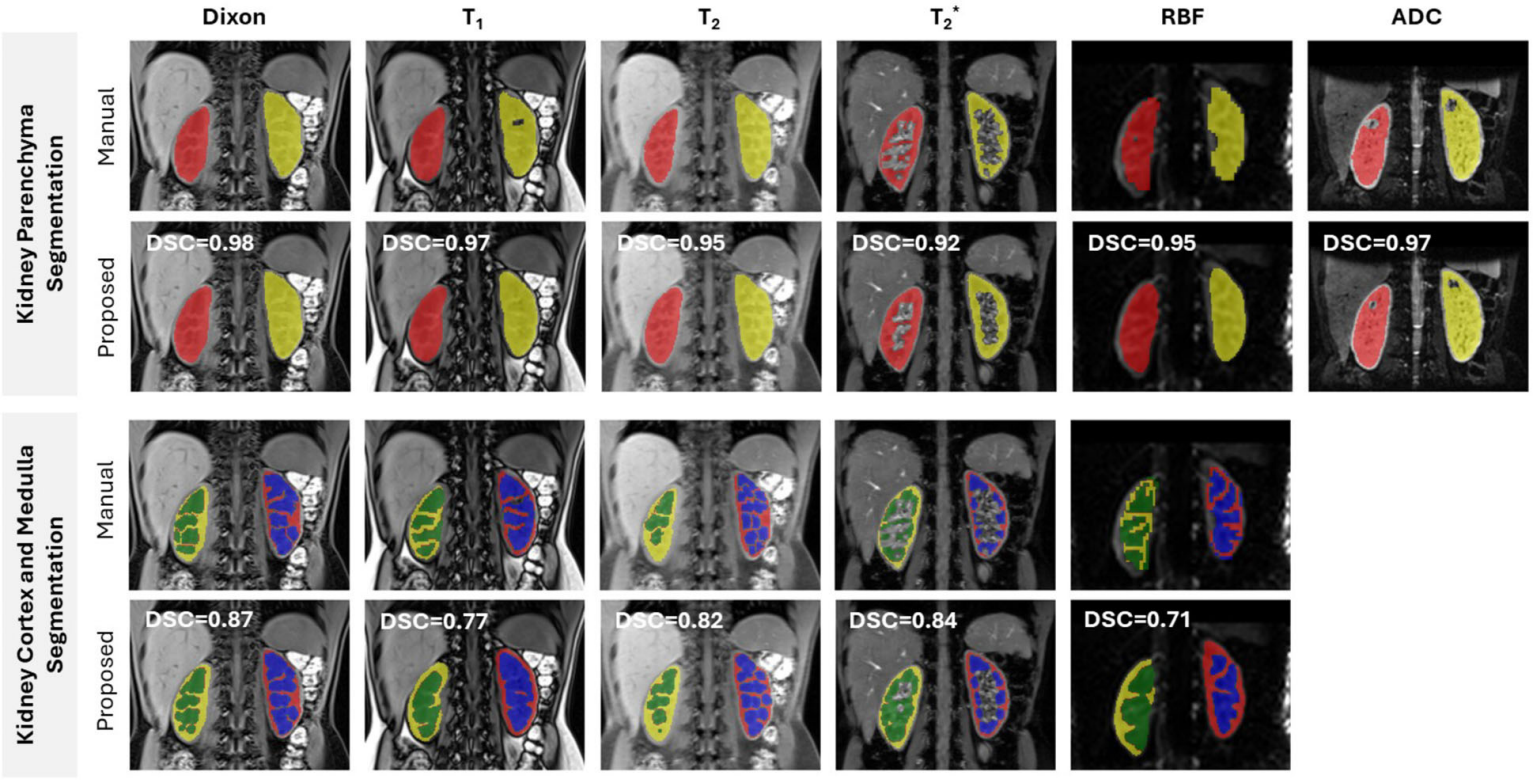
Comparison of manual and automated segmentation masks for multi‐parametric renal MR images, including Dixon, $T_{1}$, $T_{2}$, $T_{2}^{*}$, RBF, and ADC data. The results show segmentation for the left and right kidneys (top) and corresponding cortex and medulla (bottom) in a healthy subject. Automated results closely match the manual kidney parenchyma segmentations with sharp boundaries. Dixon‐based segmentation demonstrates the highest agreement with manual cortex and medulla masks, capturing fine anatomical details, whereas other contrasts, particularly RBF, exhibit larger discrepancies in cortico‐medullary transitions.

Figure 3 illustrates examples of segmented images comparing manual and automated segmentation masks for a healthy subject. The model achieved high‐fidelity kidney parenchyma segmentation across all contrasts, closely approximating manual annotations. Dixon‐based predictions provided superior cortex–medulla delineation and preserved fine anatomical structures, while high‐contrast images ($T_{1}$, $T_{2}$, $T_{2}^{*}$) also yielded high‐fidelity segmentations. However, limitations were observed, primarily due to under‐segmentation of thin cortical regions and over‐segmentation of cortex‐medullary transitions. Furthermore, low‐contrast regions, especially RBF images, exhibited increased segmentation uncertainty. Automated predictions generally exhibited smoother, more continuous boundaries compared to the occasionally irregular and fragmented contours observed in manual delineations. Additional qualitative results are provided in Figures S4 and S5, showing the best, median, and worst representative cases.

**FIGURE 4:**
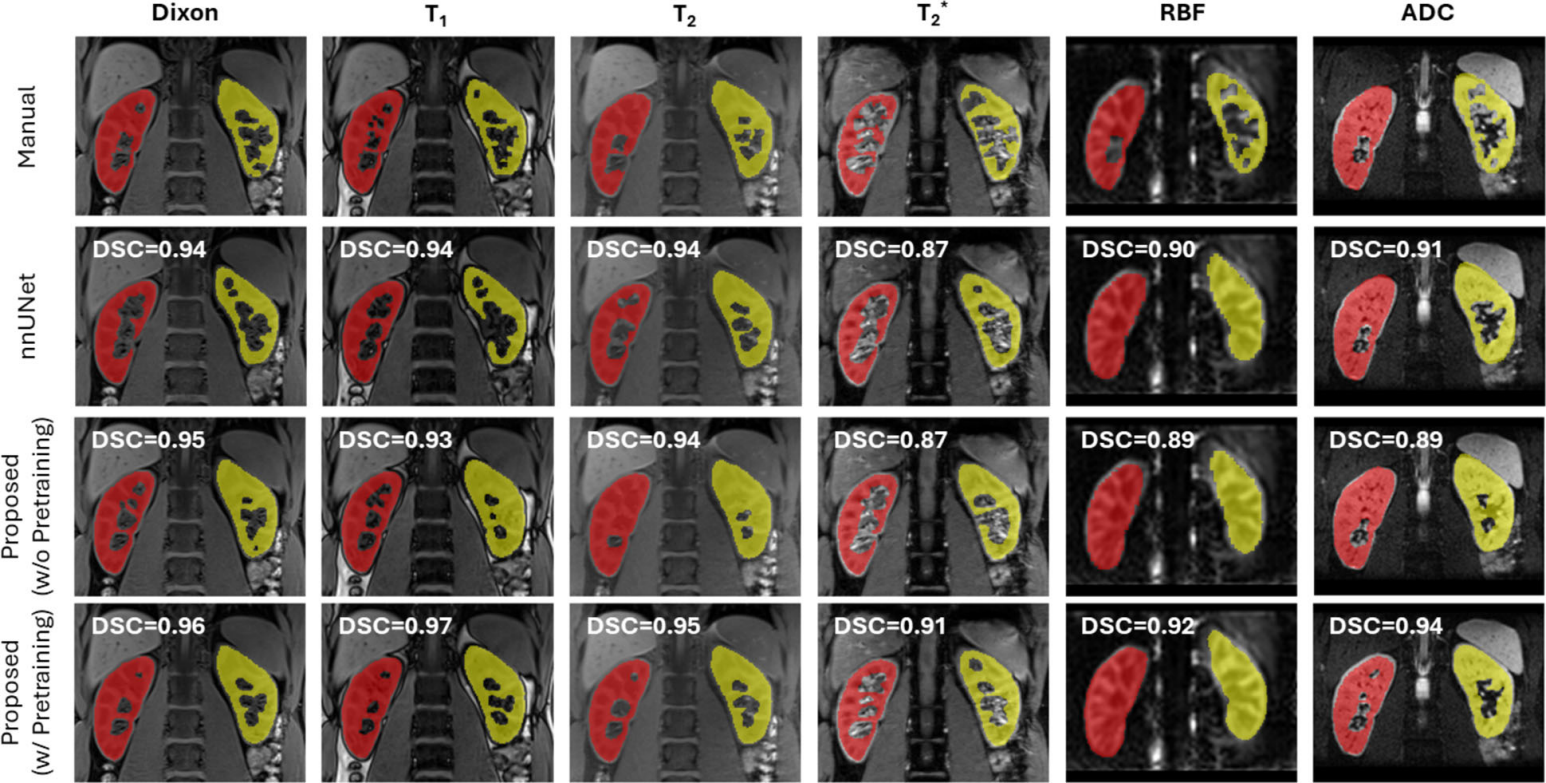
Qualitative comparison of kidney segmentation from different methods across various contrasts (Dixon, $T_{1}$, $T_{2}$, $T_{2}^{*}$, RBF, and ADC) in a healthy subject. The rows show results for different segmentation methods: Manual, nnU‐Net [19], the proposed method without contrastive learning pre‐training (ablation), and with pre‐training. Our segmentation strategy with pre‐training consistently produces segmentation masks that are more accurate and qualitatively aligned with the manual ground truth across all contrasts compared to the other comparative approaches.

We compared the proposed segmentation network, with and without contrastive pre‐training, against nnU‐Net [19] (Table S1). For high‐contrast images (Dixon, $T_{1}$ and $T_{2}$), nn‐UNet and the proposed method attained similar mean DSC values, although our approach had lower standard deviations. The improvements over nn‐UNet were most pronounced in low‐contrast RBF images, with relative DSC gains of +14% for the kidney, +12% for the cortex, and +30% for the medulla. Removing contrastive pre‐training caused notable relative drops in performance, especially for RBF images (kidney: −11%, cortex: −12%, medulla: −25%). Qualitative results (Figures 4 and S6) indicate that pre‐training improved boundary delineation and consistency compared to nnU‐Net and the randomly initialized model. Performance across different available annotated subjects of 6, 12, and 26 (Table S2) shows that pretraining significantly improves segmentation performance compared to random initialization.

### 3.2 Motion Correction Performance

We compared affine registration, affine combined with non‐rigid registration, and a no‐registration baseline (w/o registration) to evaluate the efficacy of motion correction. The quantitative results of the five‐fold cross‐validation are summarized in Table 2 The proposed registration method consistently achieved superior alignment compared to the unregistered series. Significant improvements were observed in the image similarity metrics $D_{\mathrm{MI}}$ ($p_{\text{affine}}=0.002$, $p_{\text{affine}+\text{non}-\text{rigid}}<0.001$) and $D_{\mathrm{PCA}}$ ($p_{\text{affine}}=0.01$, $p_{\text{affine}+\text{non}-\text{rigid}}<0.001$). Affine registration improved alignment, with non‐rigid registration providing further metric enhancements. Additionally, the low percentage of negative pixels in the Jacobian determinant $|J_{u}|\leq 0$ suggested robust and physically plausible transformations.

**TABLE 2:** Quantitative results for image registration from Dixon to baseline contrasts ($T_{1}$, $T_{2}$, $T_{2}^{*}$, RBF, and ADC) evaluated using five‐fold cross‐validation.

Metrics	Parameter	w/o Registration	Affine registration	Affine + nonrigid registration
$D_{\mathrm{NMI}}$	All	0.22 ± 0.09	0.31 ± 0.02	**0**.**32** ± **0**.**02**
$D_{\mathrm{PCA}}$		2.64 ± 0.09	2.63 ± 0.08	**2**.**62** ± **0**.**08**
% of $|J_{u}|\leq 0$		—	—	0.01 ± 0.02
${\mathrm{DSC}}_{\text{warp}-\text{volume}}$	$T_{1}$ map	0.78 ± 0.03	0.79 ± 0.04	**0**.**81** ± **0**.**03**
	$T_{2}$ map	0.81 ± 0.03	0.82 ± 0.03	**0**.**84** ± **0**.**04**
	$T_{2}^{*}$ map	0.79 ± 0.05	0.80 ± 0.04	**0**.**84** ± **0**.**05**
	RBF	0.59 ± 0.11	0.73 ± 0.14	**0**.**74** ± **0**.**13**
	ADC	0.74 ± 0.13	**0**.**79** ± **0**.**04**	**0**.**79** ± **0**.**04**
${\mathrm{DSC}}_{\text{warp}-\text{cortex}}$	RBF	0.42 ± 0.09	0.63 ± 0.07	**0**.**64** ± **0**.**07**
${\mathrm{DSC}}_{\text{warp}-\text{medulla}}$	RBF	0.45 ± 0.09	0.65 ± 0.05	**0**.**66** ± **0**.**05**

*Note*: Metrics include the normalized Mutual Information ($D_{\mathrm{NMI}}$), PCA dissimilarity ($D_{\mathrm{PCA}}$), the percentage of pixels with non‐positive Jacobian determinant (% of $|J_{u}|\leq 0$), and Dice similarity coefficient (${\mathrm{DSC}}_{\text{warp}}$). Evaluations are presented for no registration, affine registration, and affine + nonrigid registration for the kidney volume and the cortex and medulla in RBF. Bold values indicate the best performance achieved for each metric at a given parameter setting.

The proposed registration demonstrated improved segmentation overlap across all imaging contrasts compared to unregistered masks. ${\mathrm{DSC}}_{\text{warp}}$ values (warped mask to manual mask of target contrast) for the kidney parenchyma increased with registration, but the segmentation network (predicted contrast mask to manual contrast mask) provided a stronger agreement. Furthermore, we observed significant improvements between affine and affine plus non‐rigid registration for $T_{2}$ and $T_{2}^{*}$ (p<0.001) and RBF images (p=0.003). However, minimal differences were found between affine and affine plus non‐rigid registration in ADC images (p=0.06). For cortex and medulla segmentation in RBF images, the registration‐based method achieved significantly higher DSC values (p<0.001) compared to automated segmentation. This enhancement resulted in a DSC increase of 12% for the cortex and 17% for the medulla compared to the automated segmentation. We achieved optimal renal multi‐parametric MRI segmentation by leveraging the combined capabilities of automated segmentation and registration. Segmentation demonstrated robust performance on high‐contrast images, while registration facilitated the accurate transfer of these segmentations to low‐contrast images.

Figure S7 presents quiver plots visualizing the estimated motion, along with transformed kidney parenchyma segmentation contours overlaid on the moving images for $T_{1}$, $T_{2}$, $T_{2}^{*}$, RBF, and ADC of a patient with prostate cancer. Results demonstrated that the proposed network successfully adapted the segmentation mask across different contrasts, even in low‐contrast RBF and ADC images. Affine registration provided global alignment by correcting mainly for translation, rotation, and scaling. The subsequent non‐rigid registration smoothed mask boundaries and accommodated local deformations.

Figure S8 presents a qualitative comparison of segmentation with and without registration for a second patient with prostate cancer and renal tumor across multiple slices. Registration enhances overall anatomical alignment and boundary fidelity across contrasts, yielding visibly tighter segmentation contours. While ADC remains challenging in a few slices, the method consistently provides clearer contour placement despite tumor‐related anatomical distortion.

**FIGURE 5:**
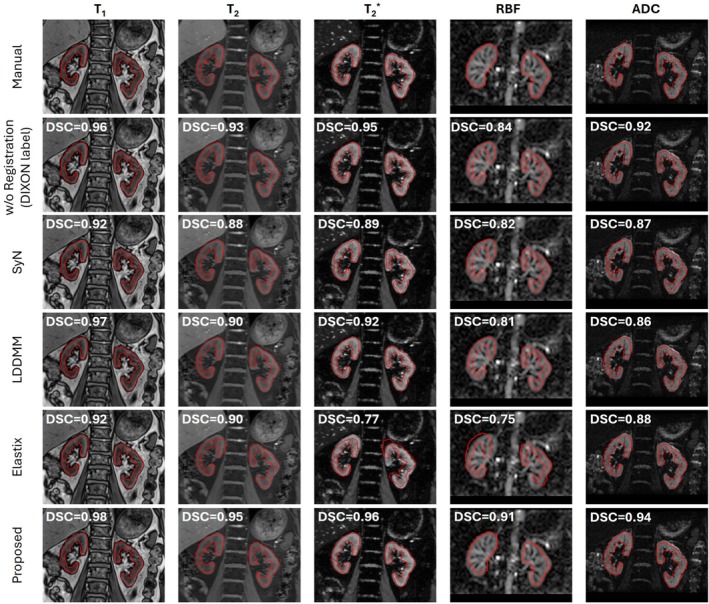
Qualitative comparison of kidney segmentation obtained using different registration methods in a patient with a neuroendocrine tumor. Red outlines indicate segmentation boundaries obtained by transforming the manual Dixon label with the estimated motion. Segmentation masks are shown for $T_{1}$, $T_{2}$ $T_{2}^{*}$, RBF, and ADC contrasts. Rows correspond to no registration, SyN [35], LDDMM [36], Elastix [37], and the proposed registration. Conventional registration methods show misalignments despite extensive hyperparameter tuning. The proposed method produces segmentation masks most consistent with manual annotations across all contrasts.

Conventional image registrations (SyN [35], LDDMM [36], and Elastix [37]) often yielded lower DSC and Mutual Information than the unregistered Dixon mask (Table S3). Contrarily, our registration approach consistently improved alignment across all contrasts and achieved an increase in DSC of 29%–37% in RBF and of 29%–37% in ADC images, while maintaining minimal folding in the deformation fields. Qualitative assessment (Figure 5) confirmed smoother and more accurate kidney boundary alignment, particularly in RBF images where conventional methods failed. Quiver plots (Figure S9) further demonstrated coherent displacement fields without isolated clusters or abrupt jumps, unlike other competing methods.

Table S4 summarizes the quantitative evaluation of registration performance across the 2D, 2.5D, and 3D architectures. The 2.5D model showed marginal numerical gains in NMI and ADC Dice, ranging between 0.01 and 0.02, but these differences were not statistically significant and did not translate into improved cortical or medullary overlap relative to the 2D approach. The 3D U‐Net demonstrated the lowest overall performance, with pronounced degradation for contrasts exhibiting sparse or single‐slice coverage (e.g., $T_{2}$, $T_{2}^{*}$). This behavior reflects the difficulty of learning spatially coherent 3D deformations from volumes with extreme voxel anisotropy and limited through‐plane contextual information. Our proposed 2D approach is computationally efficient and provided the most reliable registration across contrast types.

### 3.3 Evaluation of Volume Measurements

**FIGURE 6:**
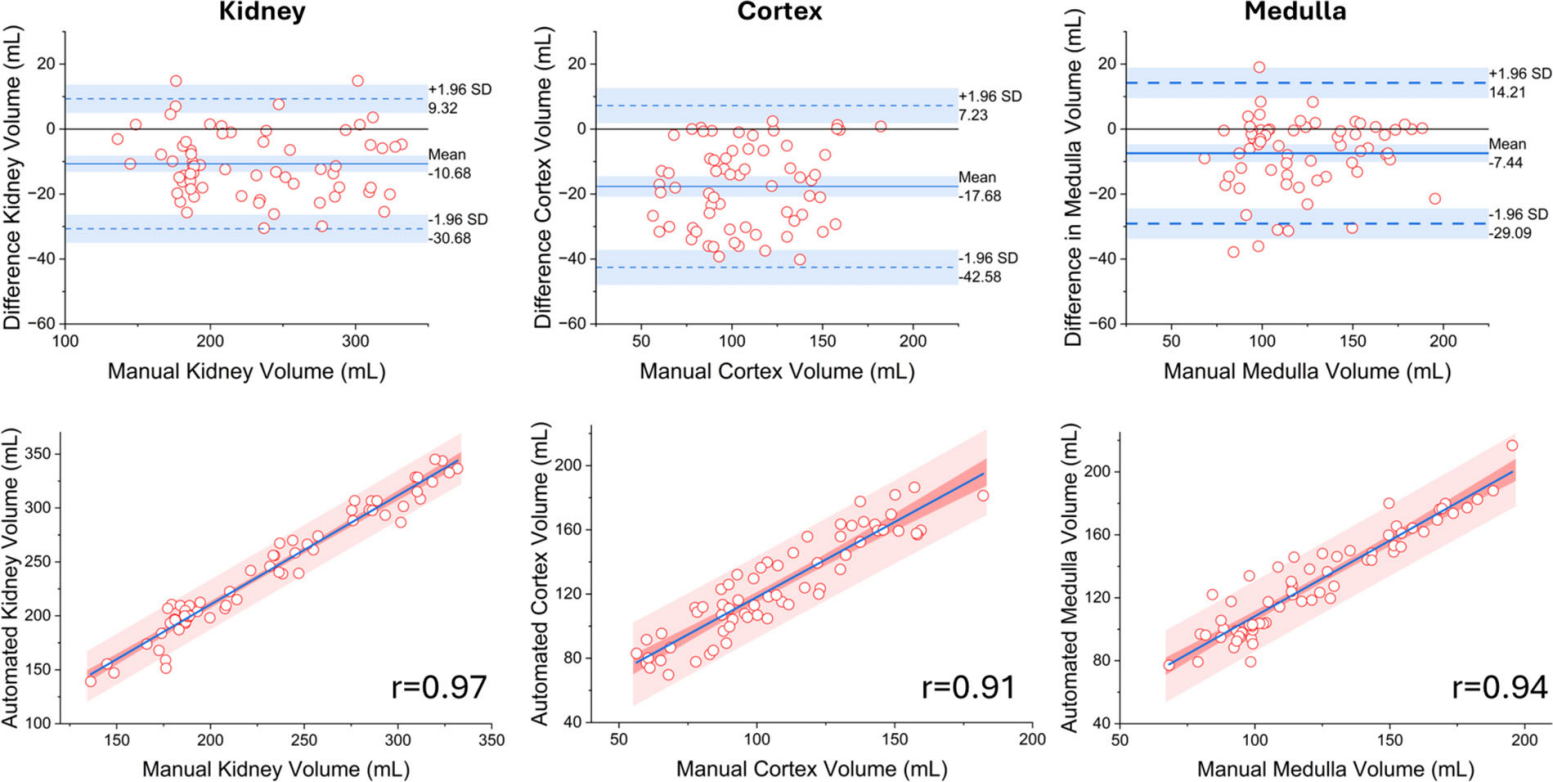
Comparison of manually and automatically generated volume measurements for the total kidney, cortex, and medulla, obtained through five‐fold cross‐validation on Dixon images. (Top) Modified Bland–Altman plots display the mean difference (bias) as a solid blue line and the limits of agreement (mean difference ±1.96 standard deviation (SD)) as dashed blue lines. The plots reveal a negative bias, indicating that the automated method underestimates volumes compared to manual measurements, with most data points lying within the agreement limits. (Bottom) Regression plots show the linear fit (solid blue line), the 95% confidence interval (dark red shading), and the prediction interval (light red shading). These plots demonstrate strong correlations between automated and manual measurements (*r* > 0.9), confirming that the proposed segmentation network provides volumetric estimates closely matching manually obtained volumes.

Figure 6 illustrates the agreement between manually measured and predicted total kidney, cortical, and medullary volumes derived from Dixon images using five‐fold cross‐validation. The modified Bland–Altman analyses showed that the automated method exhibited a negative bias (kidney: −9.32 mL, cortex: −7.20 mL, medulla: −1.21 mL), with most observations within the limits of agreement and no systematic trends. Regression analyses revealed strong linear correlations between manual and automated measurements ($r_{\text{kidney}}=0.97$, $r_{\text{cortex}}=0.91$, $r_{\text{medulla}}=0.94$), with narrow 95% confidence and prediction intervals. Scatter points clustered more tightly around the regression line for the kidney volume compared to the cortex and medulla volumes.

### 3.4 Feature Extraction and Quantitative Analysis

**FIGURE 7:**
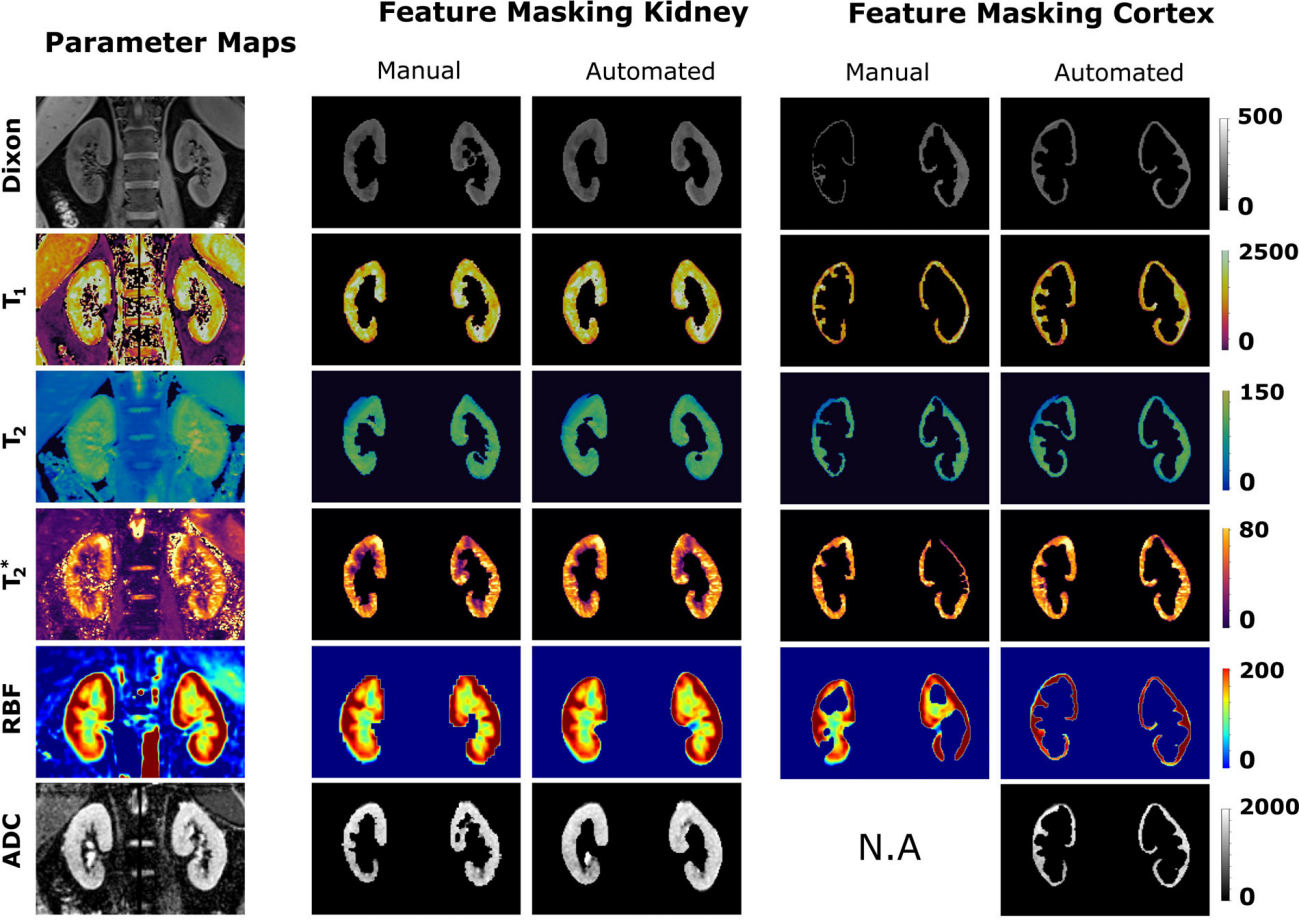
Masked multi‐parametric maps of kidney parenchyma and cortex for Dixon, $T_{1}$, $T_{2}$, $T_{2}^{*}$, RBF, and ADC data of a patient with prostate cancer. Comparison between predicted and manual delineations demonstrates feature capture accuracy. N.A. indicates unavailable manual segmentations for ADC cortex due to low contrast.

Manual and automated segmentation were used to extract features from parametric maps. The complete processing pipeline required approximately 15 s per scan on a single GPU, while parallel execution on 6 CPU cores completed in roughly 70 s per scan. Figure 7 shows masked maps for the kidney parenchyma and cortex to compare the agreement between both methods. The automated approach successfully captured key features across different imaging contrasts, closely matching manual segmentation. Image registration further improved cortex segmentation for RBF images and enabled the segmentation of diffusion‐weighted images, which are difficult to segment manually due to poor contrast.

Results from five‐fold cross‐validation are presented separately for patients and volunteers with values for the kidney parenchyma, cortex, and medulla (Table 3). For patients, the automated measurements closely matched the manual reference across all imaging modalities, exhibiting minimal bias and strong agreement. High intra‐class correlation coefficients, ranging from 0.95 to 0.99, confirmed the strong reliability of the automated pipeline for the entire kidney as well as the cortex and medulla evaluations. For healthy subjects, agreement between manual and automated measures remained high overall, though greater variability in group. Lower ICC measures were observed especially in $T_{1}$ maps (${\mathrm{ICC}}_{T_{1}-\text{Kidney}}$ = 0.81, ${\mathrm{ICC}}_{T_{1}-\text{Cortex}}$ = 0.76) and $T_{2}^{*}$ maps (e.g., ${\mathrm{ICC}}_{T_{2}-\text{Cortex}}$ = 0.78), indicating increased variability. Nevertheless, consistency remained high in RBF and ADC estimates across all regions, with ICC values exceeding 0.85.

**TABLE 3:** Overview of manual versus automated measures for the multiparametric scan protocol feature extraction.

			Manual	Automated		
Parameter	Structure	Unit	Mean ± SD	Mean ± SD	Bias	ICC
Patients						
$T_{1}$	Kidney	ms	1630.75 ± 208.84	1637.75 ± 200.38	7.01	0.99 [0.98, 0.99]
	Cortex		1505.23 ± 218.92	1484.50 ± 226.01	−20.73	0.98 [0.95, 0.99]
	Medulla		1694.75 ± 205.33	1726.75 ± 199.96	32.0	0.97 [0.87, 0.99]
$T_{2}$	Kidney	ms	85.63 ± 19.51	85.26 ± 18.78	−0.37	0.99 [0.98, 0.99]
	Cortex		87.25 ± 26.59	87.01 ± 25.41	−0.24	0.99 [0.99, 1]
	Medulla		83.80 ± 16.25	84.65 ± 18.32	0.85	0.96 [0.93, 0.98]
$T_{2}^{*}$	Kidney	ms	47.25 ± 11.09	47.55 ± 11.42	0.3	0.99 [0.98, 0.99]
	Cortex		48.18 ± 12.30	49.10 ± 13.57	0.91	0.98 [0.97, 0.99]
	Medulla		44.97 ± 10.09	44.02 ± 9.26	−0.95	0.96 [0.92, 0.98]
RBF	Kidney	mL/100 g/min	110.94 ± 46.56	107.67 ± 43.20	−3.27	0.97 [0.95, 0.98]
	Cortex		120.64 ± 55.57	114.93 ± 53.54	−5.71	0.97 [0.94, 0.99]
	Medulla		101.25 ± 39.79	95.81 ± 35.57	−5.45	0.95 [0.9, 0.98]
ADC	Kidney	$10^{-6}{\mathrm{mm}}^{2}$/s	1256.82 ± 239.85	1247.73 ± 233.43	−9.09	0.99 [0.99, 1]
Healthy subjects						
$T_{1}$	Kidney	ms	1683.15 ± 118.49	1604.56 ± 144.48	−36.42	0.81 [0.75, 0.9]
	Cortex		1538.76 ± 115.03	1495.73 ± 152.46	−28.79	0.76 [0.67, 0.94]
	Medulla		1752.87 ± 121.92	1699.71 ± 160.46	−39.28	0.78 [0.67, 0.9]
$T_{2}$	Kidney	ms	88.42 ± 5.14	88.32 ± 5.34	−0.11	0.96 [0.88, 0.99]
	Cortex		85.76 ± 4.85	84.74 ± 4.59	−1.02	0.93 [0.73, 0.95]
	Medulla		90.05 ± 6.21	90.23 ± 6.09	0.19	0.98 [0.96, 0.99]
$T_{2}^{*}$	Kidney	ms	50.77 ± 3.16	51.42 ± 3.15	0.65	0.93 [0.77, 0.97]
	Cortex		55.07 ± 3.62	57.07 ± 3.12	2.01	0.78 [0.72, 0.86]
	Medulla		47.35 ± 2.92	46.56 ± 2.63	−0.79	0.88 [0.71, 0.96]
RBF	Kidney	mL/100 g/min	179.20 ± 33.98	177.54 ± 29.47	−1.65	0.92 [0.81, 0.97]
	Cortex		210.64 ± 39.02	201.19 ± 38.42	−9.45	0.86 [0.76, 0.95]
	Medulla		158.47 ± 32.68	154.69 ± 30.05	−3.78	0.96 [0.9, 0.99]
ADC	Kidney	$10^{-6}{\mathrm{mm}}^{2}$/s	1572.89 ± 61.33	1556.56 ± 54.14	−16.33	0.88 [0.72, 0.96]

The impact of our proposed 2D registration on functional quantification was evaluated by comparing mean RBF measurements derived from Dixon masks propagated with 2D, 2.5D, and 3D networks against manual masks (Table S5). In patients and healthy subjects, 2D registration produced mean RBF values close to manual measurements, with smaller biases (bias_patients_ = −3.27 mL/100 g/min, bias_healthy_ −1.65 mL/100 g/min) and higher ICC (ICC_patients_ = 0.97, ICC_healthy_ = 0.92) compared to 3D, and comparable performance to 2.5D.

## 4 Discussion

This study introduces a comprehensive postprocessing pipeline for multi‐parametric renal MRI analysis that combines deep learning‐based segmentation with registration. Together, these models generate co‐registered segmentation masks across multiple non‐contrast MR maps, enabling efficient volumetric measurement and feature extraction. The pipeline was evaluated on kidney parenchyma, cortex, and medulla, in a cohort of patients with prostate cancer or neuroendocrine tumors and healthy subjects. By reducing processing time to seconds and eliminating reader‐dependent manual effort, the method addresses a major barrier to clinical adoption of renal fMRI. Unlike earlier approaches limited to single imaging contrasts [20, 21], our framework accommodates diverse parameter maps, including low‐contrast modalities, thereby facilitating timely and informed clinical decision‐making.

The proposed segmentation achieved high fidelity in whole‐kidney delineation, closely approximating manual segmentation across multiple contrasts. High DSC and low HD values underscore its robustness in capturing renal anatomy independent of contrast type. Performance was strongest in Dixon and $T_{1}$ maps due to their superior tissue contrast, while lower accuracy in RBF reflected the reduced medullary signal‐to‐noise ratio.

Segmentation of the cortex and medulla proved more challenging, consistent with the low inter‐reader agreement reported in manual delineation [20, 46, 58]. This difficulty arises from inherent ambiguity at the cortico‐medullary boundary, particularly in low‐contrast maps such as RBF and ADC. To address this, we implemented a registration‐based propagation strategy to transfer high‐quality Dixon masks to lower‐contrast images. This strategy substantially improved RBF segmentation and, despite the absence of ADC ground truth, parenchymal DSC values indicated reliable performance. These results demonstrate that leveraging information across parameter maps effectively mitigates the limitations posed by low contrast in individual contrasts.

The evaluation of image registration results is usually challenging because no ground truth is available for definitive comparisons. However, improvements in similarity metrics and landmark overlap confirm the utility of our approach. Moreover, the superior performance of non‐rigid over affine registration highlights the capacity to correct for local deformations and accommodate anatomical variability across different contrasts and patient conditions.

Our quantitative results demonstrate that the 2D registration framework achieved the best overall alignment and produced the most reliable mean RBF values compared to 2.5D and 3D approaches. This superiority stems from a fundamental physical constraint: the through‐plane resolution (3–6 mm slice thickness) is comparable to or greater than typical respiratory motion (3–4 mm [59]). Consequently, sub‐resolution out‐of‐plane motion cannot be recovered, making slice‐to‐slice uncertainty an acquisition‐defined limitation. This explains the modest gain of 2.5D and the poorer generalization of 3D U‐Nets. For volumetric features like mean RBF, computed via per‐slice integration, accurate in‐plane alignment is thus the dominant and optimizable factor. Consequently, small in‐plane boundary errors have a larger aggregate effect on quantification than unresolvable out‐of‐plane motion, explaining why the 2D strategy outperforms the 3D model.

Baseline comparisons further emphasize the strengths of our pipeline. Contrastive pre‐training was critical for stable segmentation at ambiguous cortex–medulla boundaries and under limited annotation conditions, where nnU‐Net and randomly initialized models underperformed. Embeddings learned from $T_{2}$ and $T_{2}^{*}$ constraint maps translated into improved separability between cortex and medulla, improving segmentation across additional and lower‐contrast parametric maps. This demonstrates that the proposed segmentation captures meaningful tissue characteristics beyond the pre‐training contrasts ($T_{2}$ and $T_{2}^{*}$), yielding transferable representations that support robust multi‐parametric renal MRI analysis in heterogeneous datasets. Registration comparisons against conventional methods also showed that our method propagates masks more reliably. Together, these results demonstrate how our framework mitigates challenges of low‐contrast imaging and scarce annotations to enable robust multi‐parametric analysis through accurate segmentation and reliable mask propagation.

Quantitative feature extraction is critical for multi‐parametric MRI applications. The robust correlation between manual and automated kidney, cortex, and medulla volume estimations and derived quantitative metrics demonstrates an effective replication of manual quantification, a crucial prerequisite for clinical translation. This consistency indicates that our automated method can be reliably integrated into clinical workflows without compromising quantitative precision.

The substantial reduction in processing time compared to manual annotation enables large‐scale analysis and improves workflow efficiency, demonstrating practicality in clinical environments. Tests on CPU configurations indicate that inference remains feasible, further supporting scalability for routine clinical use. The registration framework also provides a foundation for longitudinal tracking of renal structures across sessions, opening avenues for monitoring disease progression, detecting subtle physiological changes, and integrating diverse imaging contrasts into comprehensive renal assessments. Robustness to incomplete datasets further strengthens real‐world applicability. Segmentation operates per contrast, and available images can be aligned while skipping missing contrasts. This flexibility ensures reliable outputs in clinical practice, where acquisitions are often heterogeneous or incomplete.

This study has several limitations. First, joint optimization of segmentation and registration was not adopted in this work, although multi‐task or cascaded frameworks have been shown to improve performance over independent training [60, 61]. On our dataset, end‐to‐end joint learning proved unstable and prone to overfitting. Hence, the tasks were trained independently for more robust results. We acknowledge that this choice prevents mutual feedback between registration and segmentation, which could improve anatomical consistency and boundary accuracy. To preserve training stability, future work could explore joint refinement using differentiable SVF or LDDMM‐based constraints [62, 63], cascaded training [64], or progressive joint optimization, in which one task is gradually incorporated into the loss of the other [61].

The choice of target contrast for the fixed image in registration strongly affects mask propagation outcomes. In this study, Dixon images were used as the fixed reference due to their superior anatomical contrast and reliable segmentation. In scenarios where Dixon sequences are unavailable, possible alternatives include selecting a different contrast with robust segmentation as the fixed image (e.g., $T_{1}$ images) or synthesizing a Dixon‐like contrast via image‐to‐image translation [65]. The generalizability of the current pipeline to these scenarios remains to be investigated.

Discrepancies between manual and automated results reflect model bias and the inherent subjectivity of manual annotation. Heterogeneous training data and limited cortico‐medullary contrast also contributed to outliers. To mitigate these challenges, adaptive segmentation [66, 67] and data harmonization [68] could enhance model generalization across diverse image contrasts and patient populations. Probabilistic reference masks [69] could also provide a more robust benchmark for training and evaluation.

Our evaluation was limited to a small, single‐center cohort with restricted disease types and age range. Larger, multi‐center datasets with a broader spectrum of renal abnormalities are needed to fully assess the generalizability of the framework, evaluate the stability of the registration method in complex lesions, and enable more robust hyperparameter tuning. Third, our approach relies heavily on manual annotations. To reduce this requirement, future work could integrate pseudo‐label generation [70, 71, 72] or localization‐aware pre‐training [73]. Finally, clinical translation requires prospective validation to establish the diagnostic and prognostic value of the pipeline across diverse renal diseases. Integration into routine workflows would benefit from interactive correction interfaces to review automated results, while generating expert‐verified labels for continuous model‐in‐the‐loop training [74].

## 5 Conclusion

We have successfully implemented a robust post‐processing pipeline for renal fMRI that effectively segments and aligns the kidneys within multi‐parametric MRI data. The resulting co‐registered, segmented images allow for rapid and precise volume and feature extraction, performed in seconds. This advancement streamlines analyses in clinical trials and large‐scale studies, ensuring consistency and efficiency. Serial measurements can be standardized for monitoring disease progression and treatment response, offering a powerful tool for research and clinical applications of multi‐parametric data.

## Funding

The work was supported by the Deutsche Forschungsgemeinschaft (DFG, German Research Foundation) under Germany's excellence strategy ‐ EXC 2064/1–Project number 390727645. Financial support of the clinical study was provided through the Wilhelm Sander‐Stiftung (Wilhelm Sander Foundation, Goethestraße 74, 80336 Munich, Germany) ‐ Project number 2020.143.1.

## Conflicts of Interest

Bernd Kühn is employed by Siemens Healthineers AG.

## Supporting information


**Figure S1:** Exemplary multi‐parametric input images and corresponding constraint maps for Dixon, T1, T2, T2*, RBF, and ADC. The constraint maps are generated by applying Principal Component Analysis (PCA) followed by k‐means clustering on the multi‐parametric data. These maps serve as a structural prior for the contrastive learning module. Voxels grouped into the same cluster (represented by the same gray level) are considered to share similar intensity and texture characteristics, encouraging the network to learn locally consistent and anatomically plausible feature representations across different tissue types.
**Figure S2:** UMAP Visualization of Constrained Contrastive Learning (CCL) Feature Embeddings: Impact of MRI Contrasts. The 2D UMAP projections illustrate how different MRI contrasts and their combinations influence the feature embeddings learned by the CCL framework. Without pretraining, clusters are poorly separated and intermixed. Using T2 or T2* individually yields well‐separated clusters, while T1 and Dixon result in less distinct separation. The proposed T2 + T2* combination achieves optimal cluster separation, forming four anatomical regions: right cortex (red), left cortex (blue), right medulla (green), and left medulla (purple). Adding T1, Dixon, or all contrasts introduces some overlap, indicating that T2 + T2* provides the most relevant information for region‐specific distinction.
**Figure S3:** UMAP Visualization of Constrained Contrastive Learning (CCL) Feature Embeddings: Impact of hyperparameters. The 2D UMAP projections illustrate the impact of key hyperparameters in the CCL framework. Without pretraining, clusters are poorly separated and intermixed. With the proposed CCL configuration (embedding dimension N=64, patch size p=4×4, temperature τ=0.1), feature embeddings form well‐separated clusters corresponding to four anatomical regions: right cortex (red), left cortex (blue), right medulla (green), and left medulla (purple). Additional visualizations for varying N, p, and τ confirm that this configuration organizes the feature space into semantically meaningful clusters.
**Figure S4:** Qualitative results of the proposed segmentation method on representative subjects with best, median, and worst performance, based on the average Dice Similarity Coefficient (DSC). For each case, manual ground truth annotations are compared with the proposed segmentation results. Segmentation masks are shown for Dixon, $T_{1}$, $T_{2}$, $T_{2}^{*}$, RBF, and ADC contrasts for the left and right kidneys.
**Figure S5:** Qualitative results of the proposed segmentation method on representative subjects with best, median, and worst performance, based on the average Dice Similarity Coefficient (DSC). For each case, manual ground truth annotations are compared with the proposed segmentation results. Segmentation masks are shown for Dixon, $T_{1}$, $T_{2}$, $T_{2}^{*}$, RBF, and ADC contrasts for the left and right cortex and medulla.
**Figure S6:** Qualitative comparison of kidney cortex and medulla segmentation from different methods across various contrasts (Dixon, $T_{1}$, $T_{2}$, $T_{2}^{*}$, RBF, and ADC) in a healthy subject. Rows correspond to different segmentation approaches: Manual (ground truth), nnU‐Net [19], our proposed method without pre‐training (ablation), and our proposed method with pre‐training. Segmentations from the proposed method with pre‐training show closer visual agreement with the manual ground truth for both cortex and medulla across all contrasts.
**Figure S7:** Visualization of motion estimation and warped segmentation contours to target Dixon contrast (reference/fixed image). Quiver plots illustrate the motion fields, with transformed kidney parenchyma contours overlaid on images from $T_{1}$, $T_{2}$, $T_{2}^{*}$, RBF, and ADC. The comparison includes results from no registration, affine registration, and affine plus non‐rigid registration. The proposed method effectively adapts segmentation across various contrasts, with affine registration addressing global motion and non‐rigid registration enhancing local accuracy.
**Figure S8:** Qualitative comparison of segmentation with and without (w/o) registration in a patient with prostate cancer and renal lesion. Representative coronal slices show segmentation contours of Dixon images overlaid on multi‐parametric MRI images ($T_{1}$, $T_{2}$, $T_{2}^{*}$, RBF, and ADC) before and after registration. Registration improves overall alignment and boundary accuracy, resulting in higher DSC values and visually tighter contour adherence. Performance on ADC images is occasionally degraded, reflecting the challenge of robustly registering highly motion‐ and noise‐sensitive parametric maps. These results highlight the ability of our method to maintain robust segmentation and accurate mask propagation, even in challenging pathological anatomies (e.g., in the presence of a tumor).
**Figure S9:** Qualitative comparison of kidney segmentation and corresponding motion fields from different registration methods in a patient with a neuroendocrine tumor. Red outlines indicate segmentation boundaries obtained by transforming the manual Dixon label according to the estimated motion, while quiver plots visualize the displacement fields. Results are shown for $T_{1}$, $T_{2}$, $T_{2}^{*}$, RBF, and ADC contrasts, obtained with no registration, SyN [35], LDDMM [36], Elastix [37], and the proposed registration strategy. Conventional registration methods show noticeable misalignments despite extensive hyperparameter tuning, whereas the proposed method produces segmentation masks and motion fields most consistent with manual annotations across all contrasts.
**Table S1:** Quantitative comparisons for image segmentation performance in different contrasts, measured using the Dice Similarity Coefficient (DSC) between manual and predicted masks, evaluated using five‐fold cross‐validation. The comparison includes results from the proposed approach, with and without pre‐training via contrastive learning, as well as the nnU‐Net framework [19]. For each method, mean DSC values and standard deviations are reported. An ANOVA test was performed to assess statistical significance, with *p*‐values indicating differences in performance between the proposed model and the other methods.
**Table S2:** Quantitative comparisons for image segmentation performance in different contrasts ($T_{1}$, $T_{2}$, $T_{2}^{*}$, RBF, and ADC) for the proposed approach with (w/) and without (w/o) contrastive pre‐training, where w/o corresponds to random initialization. The models were fine‐tuned on subsets of 6, 12, and 26 subjects and evaluated on a fixed test fold of six subjects. For each method, mean Dice Similarity Coefficient (DSC) values and standard deviations are reported between manual and predicted masks. An ANOVA test was performed to assess statistical significance, with p‐values indicating differences in performance between the proposed model and the randomly initialized methods.
**Table S3:** Quantitative comparisons for image registration performance from Dixon to other contrasts ($T_{1}$, $T_{2}$, $T_{2}^{*}$, RBF, and ADC) evaluated using five‐fold cross‐validation. Metrics include the Normalized Mutual Information ($D_{\mathrm{NMI}}$), the percentage of pixels with non‐positive Jacobian determinant (% of $|J_{u}|\leq 0$), and the Dice similarity coefficient (${\mathrm{DSC}}_{\text{warp}}$). Evaluations are for the kidney volume using the proposed method, SyN [35], LDDMM [36], and Elastix [37]. An ANOVA test was performed to assess statistical significance, with 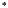 indicating p<0.05 compared to the proposed method.
**Table S4:** Quantitative results for image registration from Dixon to baseline contrasts ($T_{1}$, $T_{2}$, $T_{2}^{*}$, RBF, and ADC) evaluated using five‐fold cross‐validation. Metrics include the normalized Mutual Information ($D_{\mathrm{NMI}}$), PCA dissimilarity ($D_{\mathrm{PCA}}$), the percentage of pixels with non‐positive Jacobian determinant (% of $|J_{u}|\leq 0$), and Dice similarity coefficient (${\mathrm{DSC}}_{\text{warp}}$). Evaluations are presented for no registration, our 2D method, 2.5D slice stacking, and 3D for the kidney volume and the cortex and medulla in RBF.
**Table S5:** Comparison of mean Renal Blood Flow (RBF) measurements (mL/100 g/min) obtained from manual masks versus Dixon masks propagated with 2D, 2.5D, and 3D registration networks applied to register RBF to Dixon images.

## Data Availability

The source code is made publicly available on https://github.com/lab‐midas/Renal‐fMRI‐Framework.
