# Supplementary material for: Automated Coregistered Segmentation for Volumetric Analysis of Multiparametric Renal MRI
Source: Magn Reson Med. 2026 Feb 4;95(6):3519–35. doi: 10.1002/mrm.70288 (PMC13049282; doi:10.1002/mrm.70288)
Supplement: Supplementary file 1 — Figure S1: Exemplary multi‐parametric input images and corresponding constraint maps for Dixon, T1, T2, T2*, RBF, and ADC. The constraint maps are generated by applying Principal Component Analysis (PCA) followed by k‐means clustering on the multi‐parametric data. These maps serve as a structural prior for the contrastive learning module. Voxels grouped into the same cluster (represented by the same gray level) are considered to share similar intensity and texture characteristics, encouraging the network to learn locally consistent and anatomically plausible feature representations across different tissue types. Figure S2: UMAP Visualization of Constrained Contrastive Learning (CCL) Feature Embeddings: Impact of MRI Contrasts. The 2D UMAP projections illustrate how different MRI contrasts and their combinations influence the feature embeddings learned by the CCL framework. Without pretraining, clusters are poorly separated and intermixed. Using T2 or T2* individually yields well‐separated clusters, while T1 and Dixon result in less distinct separation. The proposed T2 + T2* combination achieves optimal cluster separation, forming four anatomical regions: right cortex (red), left cortex (blue), right medulla (green), and left medulla (purple). Adding T1, Dixon, or all contrasts introduces some overlap, indicating that T2 + T2* provides the most relevant information for region‐specific distinction. Figure S3: UMAP Visualization of Constrained Contrastive Learning (CCL) Feature Embeddings: Impact of hyperparameters. The 2D UMAP projections illustrate the impact of key hyperparameters in the CCL framework. Without pretraining, clusters are poorly separated and intermixed. With the proposed CCL configuration (embedding dimension N=64, patch size p=4×4, temperature τ=0.1), feature embeddings form well‐separated clusters corresponding to four anatomical regions: right cortex (red), left cortex (blue), right medulla (green), and left medulla (purple). Additional visualizati [file MRM-95-3519-s001.pdf]

Supporting Information

Automated Co-registered Segmentation for Volumetric Analysis of Multi-parametric Renal MRI

Aya Ghoul[1], Cecilia Liang[2], Isabelle Loster[2], Lavanya Umapathy[3], Bernd Kühn[4], Petros Martirosian[2], Ferdinand Seith[3], Sergios Gatidis[1,5], and Thomas Küstner[1]

[1]Medical Image and Data Analysis (MIDAS.lab), Department of Diagnostic and Interventional Radiology, University Hospital of Tuebingen, Germany

[2]Department of Diagnostic and Interventional Radiology, University Hospital of Tuebingen, Germany

[3]Center for Advanced Imaging Innovation and Research (CAI2R), Department of Radiology, New York University Grossman School of Medicine, USA

[4]Siemens Healthcare AG, Germany

[5]Department of Radiology, Stanford University, USA

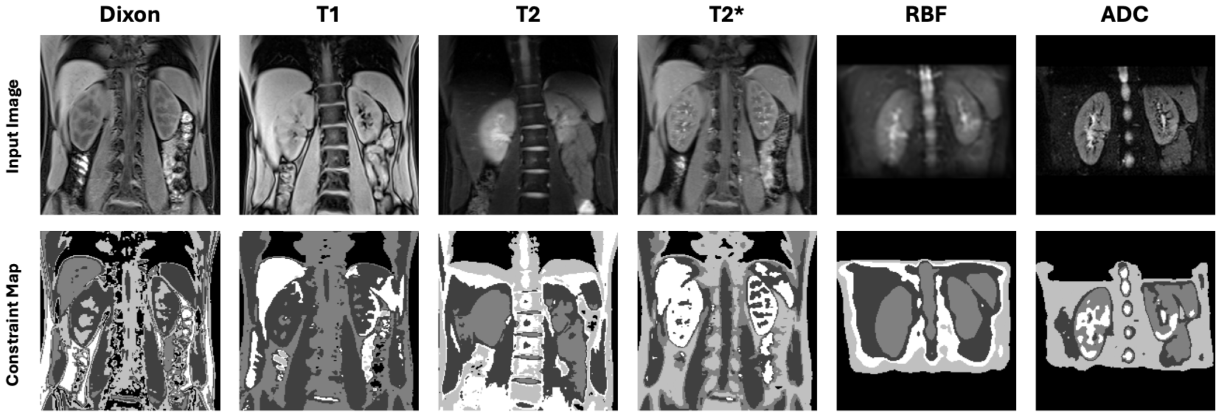

Figure S1: Exemplary multi-parametric input images and corresponding constraint maps for Dixon, T1, T2, T2*, RBF, and ADC. The constraint maps are generated by applying Principal Component Analysis (PCA) followed by k-means clustering on the multi-parametric data. These maps serve as a structural prior for the contrastive learning module. Voxels grouped into the same cluster (represented by the same gray level) are considered to share similar intensity and texture characteristics, encouraging the network to learn locally consistent and anatomically plausible feature representations across different tissue types.

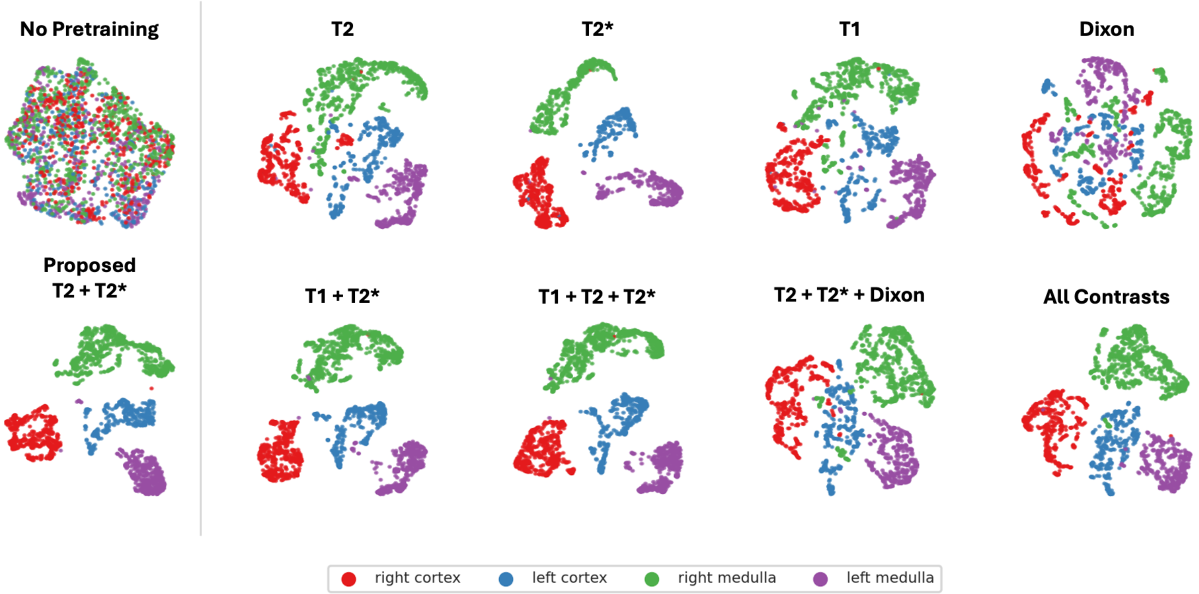

Figure S2: UMAP Visualization of Constrained Contrastive Learning (CCL) Feature Embeddings: Impact of MRI Contrasts. The 2D UMAP projections illustrate how different MRI contrasts and their combinations influence the feature embeddings learned by the CCL framework. Without pretraining, clusters are poorly separated and intermixed. Using T2 or T2* individually yields well-separated clusters, while T1 and Dixon result in less distinct separation. The proposed T2 + T2* combination achieves optimal cluster separation, forming four anatomical regions: right cortex (red), left cortex (blue), right medulla (green), and left medulla (purple). Adding T1, Dixon, or all contrasts introduces some overlap, indicating that T2 + T2* provides the most relevant information for region-specific distinction.

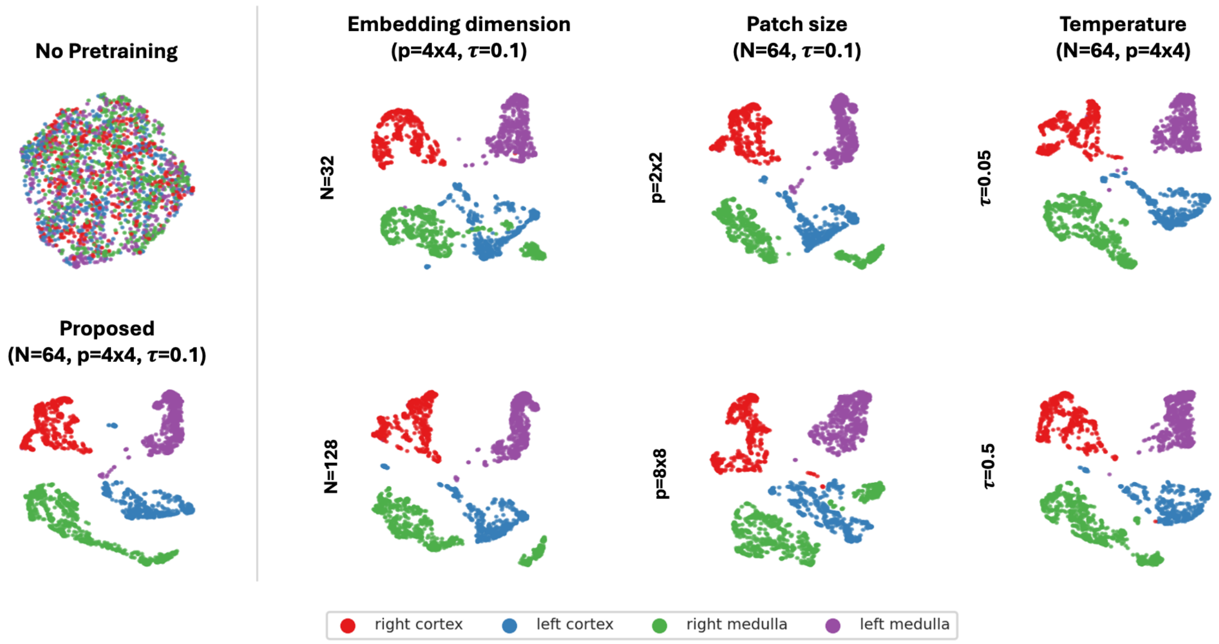

Figure S3: UMAP Visualization of Constrained Contrastive Learning (CCL) Feature Embeddings: Impact of hyperparameters. The 2D UMAP projections illustrate the impact of key hyperparameters in the CCL framework. Without pretraining, clusters are poorly separated and intermixed. With the proposed CCL configuration (embedding dimension $N = 64$, patch size $p = 4 \times 4$, temperature $\tau = 0.1$), feature embeddings form well-separated clusters corresponding to four anatomical regions: right cortex (red), left cortex (blue), right medulla (green), and left medulla (purple). Additional visualizations for varying N, p, and τ confirm that this configuration organizes the feature space into semantically meaningful clusters.

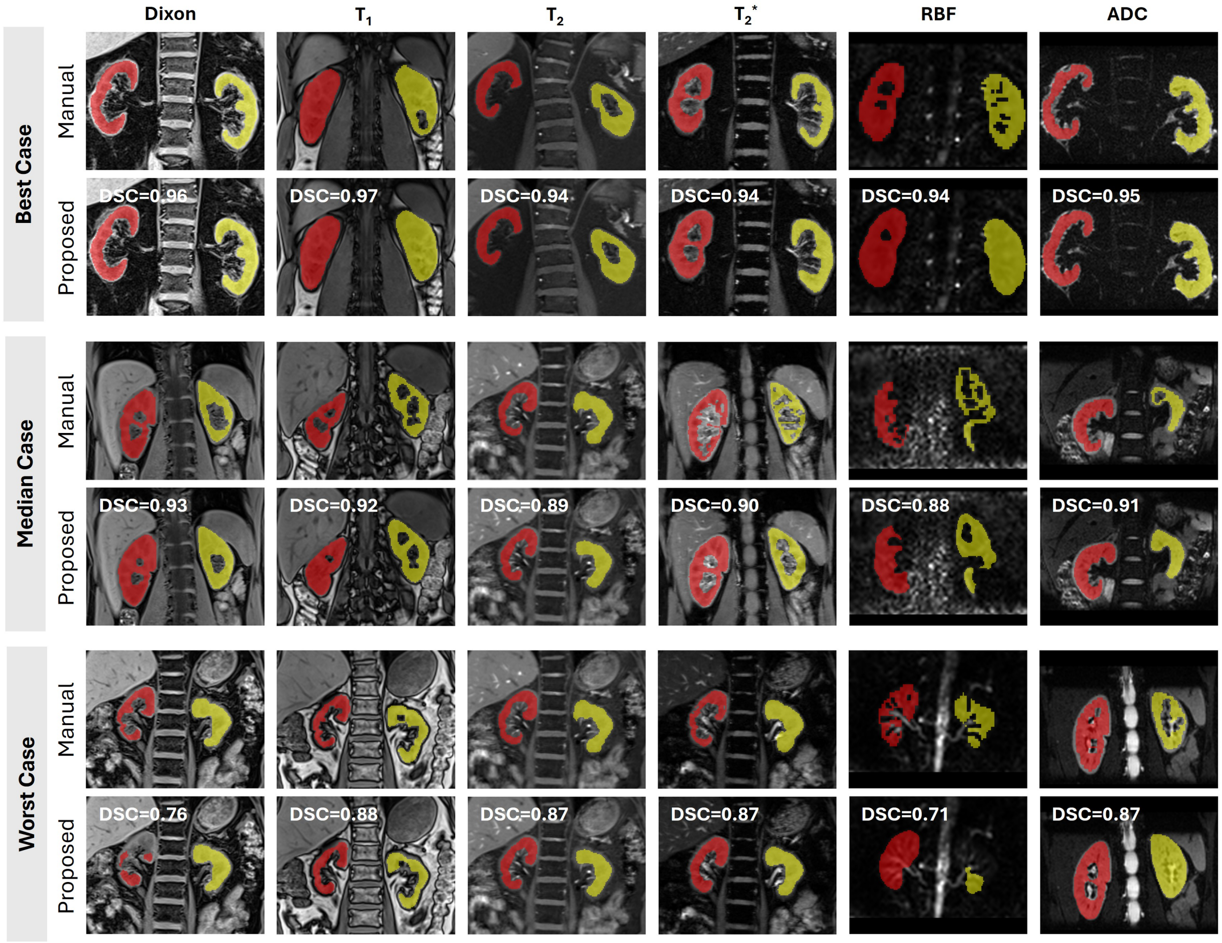

Figure S4: Qualitative results of the proposed segmentation method on representative subjects with best, median, and worst performance, based on the average Dice Similarity Coefficient (DSC). For each case, manual ground truth annotations are compared with the proposed segmentation results. Segmentation masks are shown for Dixon, T_1, T_2, T_2, RBF, and ADC contrasts for the left and right kidneys.

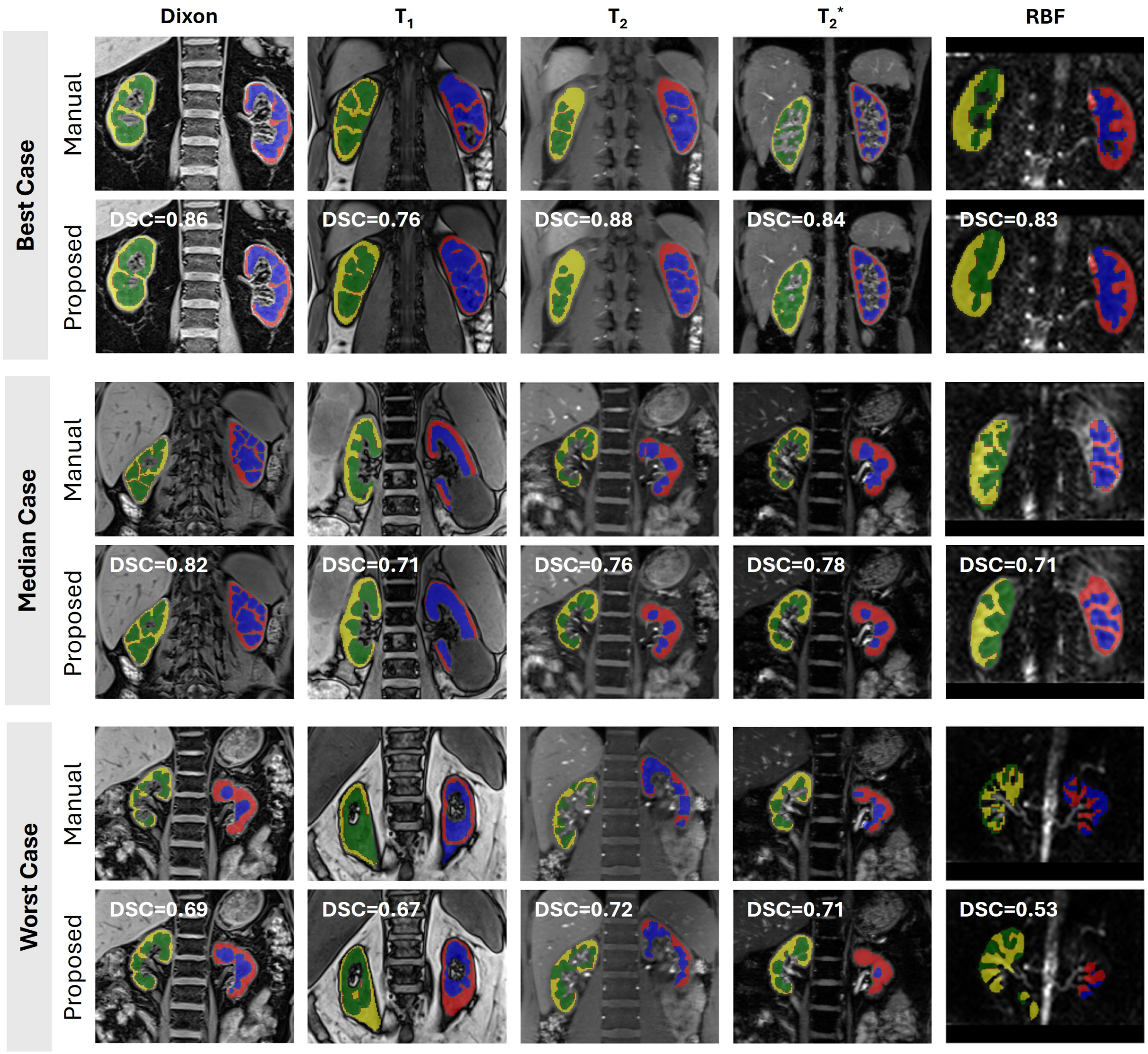

Figure S5: Qualitative results of the proposed segmentation method on representative subjects with best, median, and worst performance, based on the average Dice Similarity Coefficient (DSC). For each case, manual ground truth annotations are compared with the proposed segmentation results. Segmentation masks are shown for Dixon, T_1, T_2, T_2, RBF, and ADC contrasts for the left and right cortex and medulla.

Table S1: Quantitative comparisons for image segmentation performance in different contrasts, measured using the Dice Similarity Coefficient (DSC) between manual and predicted masks, evaluated using five-fold cross-validation. The comparison includes results from the proposed approach, with and without pre-training via contrastive learning, as well as the nnU-Net framework [1]. For each method, mean DSC values and standard deviations are reported. An ANOVA test was performed to assess statistical significance, with p-values indicating differences in performance between the proposed model and the other methods.

		proposed (w/ pre-training)	proposed (w/o pre-training)	nnU-Net
**Kidney**	Dixon	$\mathbf{0.91 \pm 0.04}$	$0.88 \pm 0.06^*$	0.91 ± 0.05
	T_1	$\mathbf{0.89 \pm 0.04}$	$0.87 \pm 0.07^*$	0.88 ± 0.05
	T_2	$\mathbf{0.89 \pm 0.04}$	$0.86 \pm 0.07^*$	0.87 ± 0.03
	T_2^*	$\mathbf{0.87 \pm 0.05}$	$0.84 \pm 0.07^*$	$0.85 \pm 0.05^*$
	RBF	$\mathbf{0.81 \pm 0.07}$	$0.71 \pm 0.09^*$	$0.72 \pm 0.22^*$
	ADC	$\mathbf{0.89 \pm 0.04}$	$0.86 \pm 0.06^*$	0.89 ± 0.05
**Cortex**	Dixon	$\mathbf{0.71 \pm 0.08}$	$0.65 \pm 0.08^*$	0.68 ± 0.08
	T_1	0.65 ± 0.06	$0.61 \pm 0.07^*$	$\mathbf{0.66 \pm 0.04}$
	T_2	$\mathbf{0.73 \pm 0.06}$	$0.69 \pm 0.09^*$	$0.72 \pm 0.14^*$
	T_2^*	$\mathbf{0.71 \pm 0.07}$	$0.66 \pm 0.11^*$	$0.65 \pm 0.14^*$
	RBF	$\mathbf{0.58 \pm 0.11}$	$0.52 \pm 0.12^*$	$0.51 \pm 0.19^*$
**Medulla**	Dixon	$\mathbf{0.79 \pm 0.05}$	$0.71 \pm 0.08^*$	0.78 ± 0.06
	T_1	$\mathbf{0.78 \pm 0.05}$	$0.69 \pm 0.08^*$	$0.76 \pm 0.05^*$
	T_2	$\mathbf{0.73 \pm 0.07}$	0.68 ± 0.06	0.73 ± 0.09
	T_2^*	$\mathbf{0.71 \pm 0.07}$	$0.64 \pm 0.08^*$	$0.65 \pm 0.19^*$
	RBF	$\mathbf{0.56 \pm 0.08}$	$0.43 \pm 0.08^*$	$0.42 \pm 0.18^*$

* For p-value < 0.05.

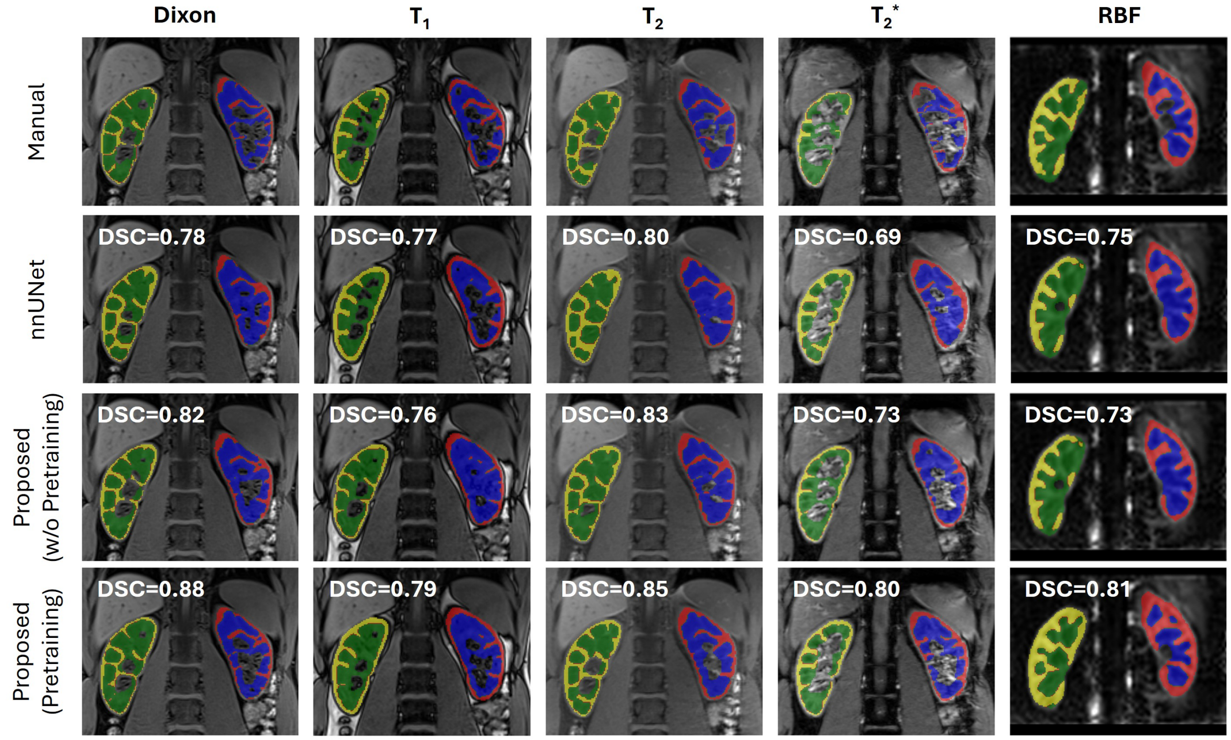

Figure S6: Qualitative comparison of kidney cortex and medulla segmentation from different methods across various contrasts (Dixon, T_1, T_2, T_2, RBF, and ADC) in a healthy subject. Rows correspond to different segmentation approaches: Manual (ground truth), nnU-Net [1], our proposed method without pre-training (ablation), and our proposed method with pre-training. Segmentations from the proposed method with pre-training show closer visual agreement with the manual ground truth for both cortex and medulla across all contrasts.

Table S2: Quantitative comparisons for image segmentation performance in different contrasts (T_1, T_2, T_2^*, RBF, and ADC) for the proposed approach with (w/) and without (w/o) contrastive pre-training, where w/o corresponds to random initialization. The models were fine-tuned on subsets of 6, 12, and 26 subjects and evaluated on a fixed test fold of six subjects. For each method, mean Dice Similarity Coefficient (DSC) values and standard deviations are reported between manual and predicted masks. An ANOVA test was performed to assess statistical significance, with p-values indicating differences in performance between the proposed model and the randomly initialized methods.

Fine-tuning subjects		**6**	**12**	**26**
Dixon	w/ pre-training	0.67 ± 0.24	0.81 ± 0.14	0.92 ± 0.03
	w/o pre-training	$0.57 \pm 0.26^*$	$0.72 \pm 0.18^*$	$0.84 \pm 0.08^*$
T_1	w/ pre-training	0.65 ± 0.28	0.76 ± 0.17	0.89 ± 0.04
	w/o pre-training	$0.56 \pm 0.3^*$	$0.69 \pm 0.21^*$	$0.87 \pm 0.07^*$
T_2	w/ pre-training	0.62 ± 0.31	0.75 ± 0.12	0.88 ± 0.03
	w/o pre-training	$0.56 \pm 0.27^*$	$0.68 \pm 0.19^*$	$0.86 \pm 0.08^*$
T_2^*	w/ pre-training	0.75 ± 0.26	0.8 ± 0.19	0.89 ± 0.03
	w/o pre-training	$0.63 \pm 0.29^*$	$0.71 \pm 0.26^*$	$0.85 \pm 0.07^*$
RBF	w/ pre-training	0.63 ± 0.26	0.74 ± 0.18	0.82 ± 0.07
	w/o pre-training	$0.55 \pm 0.32^*$	$0.63 \pm 0.24^*$	$0.72 \pm 0.11^*$
ADC	w/ pre-training	0.73 ± 0.26	0.78 ± 0.12	0.88 ± 0.03
	w/o pre-training	$0.66 \pm 0.27^*$	$0.75 \pm 0.16^*$	$0.85 \pm 0.08^*$

* For p-value < 0.05.

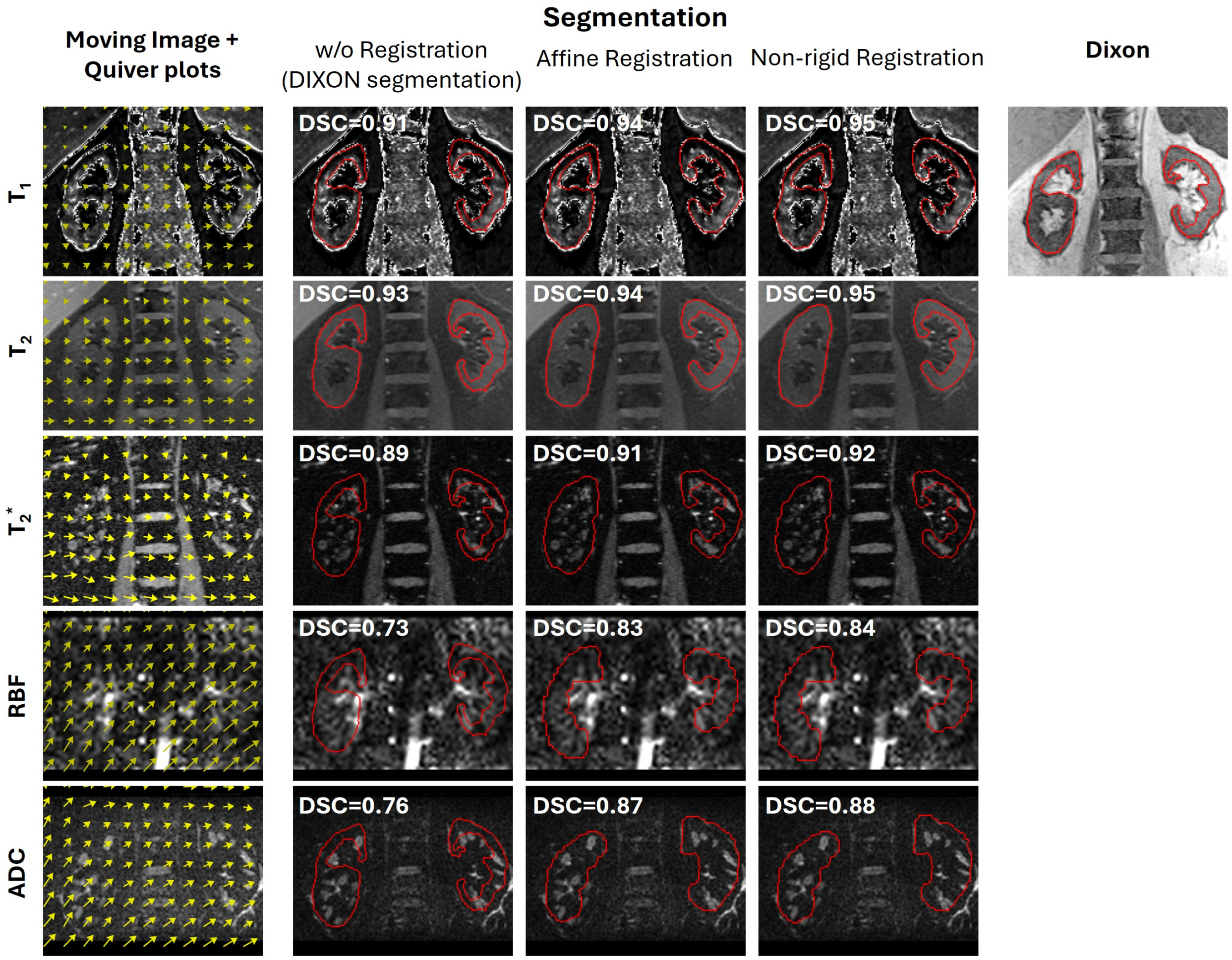

Figure S7: Visualization of motion estimation and warped segmentation contours to target Dixon contrast (reference/fixed image). Quiver plots illustrate the motion fields, with transformed kidney parenchyma contours overlaid on images from T_1, T_2, T_2^*, RBF, and ADC. The comparison includes results from no registration, affine registration, and affine plus non-rigid registration. The proposed method effectively adapts segmentation across various contrasts, with affine registration addressing global motion and non-rigid registration enhancing local accuracy.

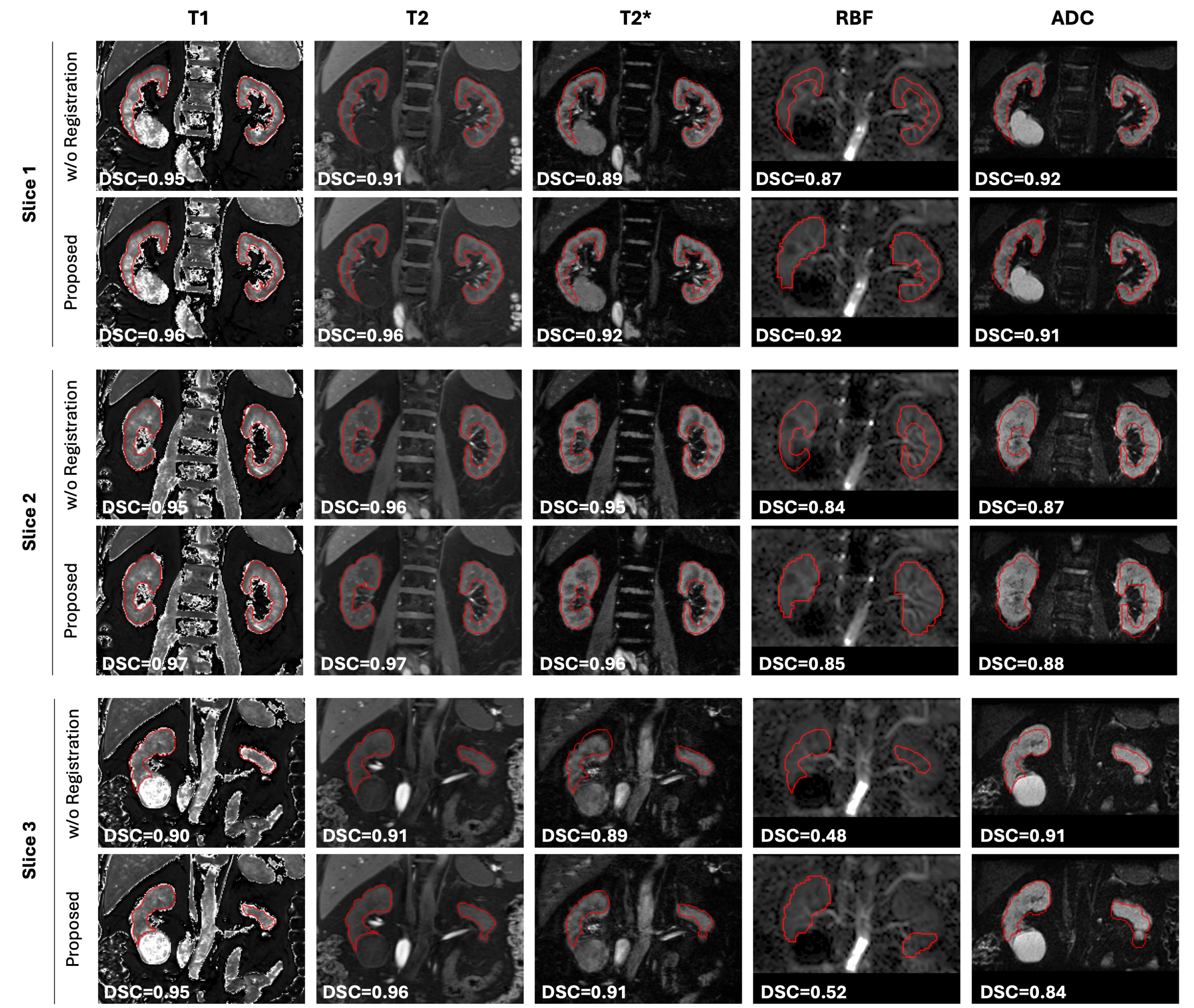

Figure S8: Qualitative comparison of segmentation with and without (w/o) registration in a patient with prostate cancer and renal lesion. Representative coronal slices show segmentation contours of Dixon images overlaid on multi-parametric MRI images (T_1, T_2, T_2^*, RBF, and ADC) before and after registration. Registration improves overall alignment and boundary accuracy, resulting in higher DSC values and visually tighter contour adherence. Performance on ADC images is occasionally degraded, reflecting the challenge of robustly registering highly motion- and noise-sensitive parametric maps. These results highlight the ability of our method to maintain robust segmentation and accurate mask propagation, even in challenging pathological anatomies (e.g., in the presence of a tumor).

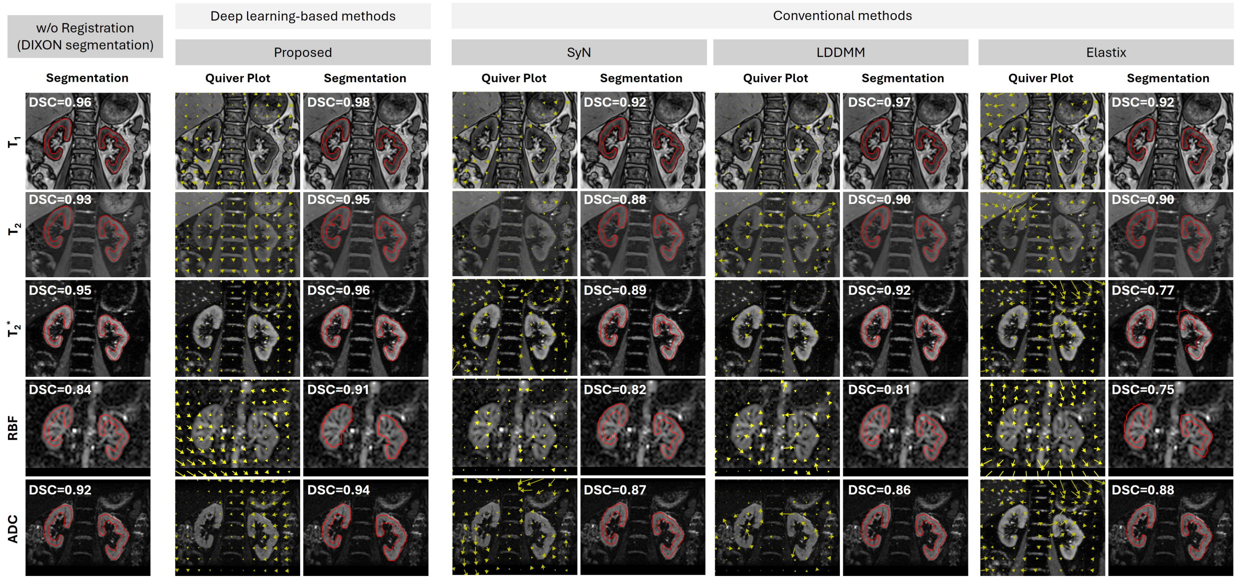

Figure S9: Qualitative comparison of kidney segmentation and corresponding motion fields from different registration methods in a patient with a neuroendocrine tumor. Red outlines indicate segmentation boundaries obtained by transforming the manual Dixon label according to the estimated motion, while quiver plots visualize the displacement fields. Results are shown for T_1, T_2, T_2^*, RBF, and ADC contrasts, obtained with no registration, SyN [2], LDDMM [3], Elastix [4], and the proposed registration strategy. Conventional registration methods show noticeable misalignments despite extensive hyperparameter tuning, whereas the proposed method produces segmentation masks and motion fields most consistent with manual annotations across all contrasts.

Table S3: Quantitative comparisons for image registration performance from Dixon to other contrasts (T_1, T_2, T_2^*, RBF, and ADC) evaluated using five-fold cross-validation. Metrics include the Normalized Mutual Information ($\mathcal{D}_{NMI}$), the percentage of pixels with non-positive Jacobian determinant (% of $|J_u| \leq 0$), and the Dice similarity coefficient (DSC_{warp}). Evaluations are for the kidney volume using the proposed method, SyN [2], LDDMM [3], and Elastix [4]. An ANOVA test was performed to assess statistical significance, with * indicating $p < 0.05$ compared to the proposed method.

		w/o Registration	Proposed	SyN	LDDMM	Elastix
All	$\mathcal{D}_{NMI}$	0.22 ± 0.09	$\mathbf{0.32 \pm 0.02}$	$0.27 \pm 0.02^*$	$0.26 \pm 0.09^*$	$0.28 \pm 0.03^*$
	$\mathcal{D}_{PCA}$	2.64 ± 0.09	$\mathbf{2.62 \pm 0.08}$	$2.69 \pm 0.11^*$	$2.68 \pm 0.14^*$	$2.69 \pm 0.12^*$
	% of $\|J_u\| \leq 0$	-	0.01 ± 0.02	$< \mathbf{0.01}^*$	$< \mathbf{0.01}^*$	$0.03 \pm 0.02^*$
DSC_{warp}	T_1 Map	0.78 ± 0.03	$\mathbf{0.81 \pm 0.03}$	$0.68 \pm 0.06^*$	$0.7 \pm 0.08^*$	$0.67 \pm 0.09^*$
	T_2 Map	0.81 ± 0.03	$\mathbf{0.84 \pm 0.04}$	$0.62 \pm 0.05^*$	$0.64 \pm 0.06^*$	$0.65 \pm 0.08^*$
	T_2^* Map	0.79 ± 0.05	$\mathbf{0.84 \pm 0.05}$	0.65 ± 0.06	$0.68 \pm 0.06^*$	$0.66 \pm 0.06^*$
	RBF	0.59 ± 0.11	$\mathbf{0.74 \pm 0.13}$	$0.45 \pm 0.14^*$	$0.38 \pm 0.16^*$	$0.37 \pm 0.12^*$
	ADC	0.74 ± 0.13	$\mathbf{0.79 \pm 0.04}$	$0.51 \pm 0.12^*$	$0.45 \pm 0.09^*$	$0.49 \pm 0.09^*$

* For p-value < 0.05.

Table S4: Quantitative results for image registration from Dixon to baseline contrasts (T_1, T_2, T_2^*, RBF, and ADC) evaluated using five-fold cross-validation. Metrics include the normalized Mutual Information ($\mathcal{D}_{NMI}$), PCA dissimilarity ($\mathcal{D}_{PCA}$), the percentage of pixels with non-positive Jacobian determinant (% of $|J_u| \leq 0$), and Dice similarity coefficient (DSC_{warp}). Evaluations are presented for no registration, our 2D method, 2.5D slice stacking and 3D for the kidney volume and the cortex and medulla in RBF.

Metrics	Parameter	w/o Registration	2D (Proposed)	2.5D	3D
$\mathcal{D}_{NMI}$	All	0.22 ± 0.09	0.32 ± 0.02	$\mathbf{0.33 \pm 0.02}$	$0.28 \pm 0.05^*$
$\mathcal{D}_{PCA}$		2.64 ± 0.09	2.62 ± 0.08	$\mathbf{2.62 \pm 0.06}$	2.65 ± 0.11
% of $\|J_u\| \leq 0$		-	0.01 ± 0.02	0.01 ± 0.02	$< \mathbf{0.01}^*$
$DSC_{warp-volume}$	T_1 Map	0.78 ± 0.03	0.81 ± 0.03	$\mathbf{0.81 \pm 0.02}$	$0.77 \pm 0.04^*$
	T_2 Map	0.81 ± 0.03	$\mathbf{0.84 \pm 0.04}$	0.83 ± 0.03	$0.77 \pm 0.06^*$
	T_2^* Map	0.79 ± 0.05	$\mathbf{0.84 \pm 0.05}$	0.83 ± 0.04	$0.75 \pm 0.06^*$
	RBF	0.59 ± 0.11	$\mathbf{0.74 \pm 0.13}$	0.73 ± 0.09	$0.61 \pm 0.12^*$
	ADC	0.74 ± 0.13	0.79 ± 0.04	$\mathbf{0.80 \pm 0.05}$	$0.75 \pm 0.07^*$
$DSC_{warp-cortex}$	RBF	0.42 ± 0.09	$\mathbf{0.64 \pm 0.07}$	0.63 ± 0.08	$0.53 \pm 0.12^*$
$DSC_{warp-medulla}$	RBF	0.45 ± 0.09	$\mathbf{0.66 \pm 0.05}$	0.66 ± 0.07	$0.54 \pm 0.07^*$

* For p-value < 0.05.

Table S5: Comparison of mean Renal Blood Flow (RBF) measurements (mL/100g/min) obtained from manual masks versus Dixon masks propagated with 2D, 2.5D, and 3D registration networks applied to register RBF to Dixon images

Group	Method	RBF	Bias	ICC
Patients	Manual	110.94 ± 46.56	0	1
	2D (proposed)	107.67 ± 43.20	$\mathbf{-3.27}$	$\mathbf{0.97}$ $[\mathbf{0.95}, \mathbf{0.98}]$
	2.5D	$\mathbf{108.51 \pm 45.84}$	-4.14	0.97 $[0.94, 0.98]$
	3D	106.20 ± 41.26	-5.42	0.95 $[0.92, 0.97]$
Healthy subjects	Manual	179.20 ± 33.98	0	1
	2D (proposed)	$\mathbf{177.54 \pm 29.47}$	-1.65	$\mathbf{0.92}$ $[\mathbf{0.81}, \mathbf{0.97}]$
	2.5D	176.83 ± 31.10	$\mathbf{-1.62}$	0.92 $[0.80, 0.95]$
	3D	171.80 ± 30.62	-7.41	0.89 $[0.78, 0.94]$

References

[1] Isensee Fabian, Jaeger Paul F, Kohl Simon AA, Petersen Jens, Maier-Hein Klaus H. nnU-Net: a self-configuring method for deep learning-based biomedical image segmentation. *Nature methods.* 2021;18(2):203–211.

[2] Avants Brian B, Epstein Charles L, Grossman Murray, Gee James C. Symmetric diffeomorphic image registration with cross-correlation: evaluating automated labeling of elderly and neurodegenerative brain. *Medical image analysis.* 2008;12(1):26–41.

[3] Beg M Faisal, Miller Michael I, Trouvé Alain, Younes Laurent. Computing large deformation metric mappings via geodesic flows of diffeomorphisms. *International journal of computer vision.* 2005;61(2):139–157.

[4] Klein Stefan, Staring Marius, Murphy Keelin, Viergever Max A, Pluim Josien PW. Elastix: a toolbox for intensity-based medical image registration. *IEEE transactions on medical imaging.* 2009;29(1):196–205.
